# Association of Obesity With COVID-19 Severity and Mortality: An Updated Systemic Review, Meta-Analysis, and Meta-Regression

**DOI:** 10.3389/fendo.2022.780872

**Published:** 2022-06-03

**Authors:** Romil Singh, Sawai Singh Rathore, Hira Khan, Smruti Karale, Yogesh Chawla, Kinza Iqbal, Abhishek Bhurwal, Aysun Tekin, Nirpeksh Jain, Ishita Mehra, Sohini Anand, Sanjana Reddy, Nikhil Sharma, Guneet Singh Sidhu, Anastasios Panagopoulos, Vishwanath Pattan, Rahul Kashyap, Vikas Bansal

**Affiliations:** ^1^ Department of Internal Medicine, Allegheny General Hospital, Pittsburgh, PA, United States; ^2^ Department of Internal Medicine, Dr. Sampurnanand Medical College, Jodhpur, India; ^3^ Department of Neurology, Allegheny General Hospital, Pittsburgh, PA, United States; ^4^ Department of Internal Medicine, Government Medical College-Kolhapur, Kolhapur, India; ^5^ Department of Immunology, Mayo Clinic, Rochester, MN, United States; ^6^ Department of Internal Medicine, Dow Medical College, Dow University of Health Sciences, Karachi, Pakistan; ^7^ Department of Gastroenterology and Hepatology, Rutgers Robert Wood Johnson School of Medicine, New Brunswick, NJ, United States; ^8^ Department of Anesthesiology and Perioperative Medicine, Mayo Clinic Rochester, MN, United States; ^9^ Department of Emergency Medicine, Marshfield Clinic, Marshfield, WI, United States; ^10^ Department of Internal Medicine, North Alabama Medical Center, Florence, AL, United States; ^11^ Department of Internal Medicine, Patliputra Medical College and Hospital, Dhanbad, India; ^12^ Department of Internal Medicine, Gandhi Medical College, Secunderabad, India; ^13^ Department of Nephrology, Mayo Clinic, Rochester, MI, United States; ^14^ Department of Gastroenterology and Hepatology, Mayo Clinic, Rochester, MI, United States; ^15^ Department of Cardiology, University of Nebraska Medical Center, Omaha, NE, United States; ^16^ Department of Medicine, Division of Endocrinology and Metabolism, State University of New York (SUNY) Upstate Medical University, Syracuse, NY, United States; ^17^ Division of Pulmonary and Critical Care Medicine, Mayo Clinic, Rochester, MI, United States

**Keywords:** obesity, COVID – 19, systematic review & meta-analysis, meta-regression analysis, severity, mortality

## Abstract

**Background:**

Obesity affects the course of critical illnesses. We aimed to estimate the association of obesity with the severity and mortality in coronavirus disease 2019 (COVID-19) patients.

**Data Sources:**

A systematic search was conducted from the inception of the COVID-19 pandemic through to 13 October 2021, on databases including Medline (PubMed), Embase, Science Web, and Cochrane Central Controlled Trials Registry. Preprint servers such as BioRxiv, MedRxiv, ChemRxiv, and SSRN were also scanned.

**Study Selection and Data Extraction:**

Full-length articles focusing on the association of obesity and outcome in COVID-19 patients were included. Preferred Reporting Items for Systematic Reviews and Meta-Analysis guidelines were used for study selection and data extraction. Our Population of interest were COVID-19 positive patients, obesity is our Intervention/Exposure point, Comparators are Non-obese vs obese patients The chief outcome of the study was the severity of the confirmed COVID-19 positive hospitalized patients in terms of admission to the intensive care unit (ICU) or the requirement of invasive mechanical ventilation/intubation with obesity. All-cause mortality in COVID-19 positive hospitalized patients with obesity was the secondary outcome of the study.

**Results:**

In total, 3,140,413 patients from 167 studies were included in the study. Obesity was associated with an increased risk of severe disease (RR=1.52, 95% CI 1.41-1.63, p<0.001, I^2^ = 97%). Similarly, high mortality was observed in obese patients (RR=1.09, 95% CI 1.02-1.16, p=0.006, I^2^ = 97%). In multivariate meta-regression on severity, the covariate of the female gender, pulmonary disease, diabetes, older age, cardiovascular diseases, and hypertension was found to be significant and explained R^2^ = 40% of the between-study heterogeneity for severity. The aforementioned covariates were found to be significant for mortality as well, and these covariates collectively explained R^2^ = 50% of the between-study variability for mortality.

**Conclusions:**

Our findings suggest that obesity is significantly associated with increased severity and higher mortality among COVID-19 patients. Therefore, the inclusion of obesity or its surrogate body mass index in prognostic scores and improvement of guidelines for patient care management is recommended.

## Introduction

The entire world is enduring the effects of the global coronavirus disease 2019 (COVID-19) pandemic, which began in December 2019, when pneumonia of unknown origin was diagnosed in Hubei province, Wuhan, China (1, 2). It was later in January 2020 that the novel coronavirus strand was isolated and subsequently named severe acute respiratory syndrome coronavirus 2 (SARS-CoV-2) in February 2020 (3, 4). As of 28 December 2021 the Covid-19 pandemic has affected >281 million individuals and has led to >5.4 million global deaths (5). Even though many treatments have been proposed to combat COVID-19, there is currently no uniformly successful therapy (6–11). Although it is a widespread disease affecting multiple systems, obesity has been identified as one of the major comorbid factors in patients suffering from COVID-19 (12–21).

Overweight (BMI 25 kg/m^2^-29.9 kg/m^2^) and obesity (BMI 30 kg/m^2^ or more) are major public health problems, especially during the COVID-19 pandemic, because of their association with increased morbidity and mortality (22, 23). Berrington de Gonzalez et al. (2010) studied the association between being overweight and obesity on overall mortality in 1.46 million white adults over a median follow-up period of 10 years. They found an approximately linear relationship in the hazard ratios for BMI. The hazard ratio for every 5-unit increment of BMI was 1.31 in the BMI range of 25 kg/m^2^ to 49.9 kg/m^2^ (24). According to the 2017-2018 National Health and Nutrition Examination Survey (NHANES), approximately 42.5% of U.S. adults aged 20 or over are obese and approximately 9% have class 3 obesity or severe obesity (BMI 40 kg/m^2^or more) (25).

According to WHO, the prevalence of obesity has nearly tripled in the last four decades amounting to 13% of the entire world’s adult population (26), which is a cause for concern during the pandemic. The interplay between obesity and other disease conditions has been established for a long time. The presence of these comorbid determinants is related to increased predisposition and severity of COVID-19 (27–30). Many studies have reported increased rates of hospitalization, mechanical ventilation, and mortality in patients with a higher BMI (31–35).

During the pandemic, due to worldwide lockdowns lasting several months, compromised work routine, increased calorie intake, lack of exercise options, and stress due to uncertainty, people are at an increased risk of becoming overweight and developing obesity (36). This could have an excessive toll on the management of COVID-19 disease. To mitigate the impact of heightened morbidity and mortality associated with COVID-19 infection in patients with obesity, it is vital to be cognizant of the implications of increased BMI and its dynamic interaction with other comorbid components. Hence, we evaluated obesity as a paramount risk factor for mortality and severity in COVID-19 infection, independent of potential confounders *via* systematic review and meta-regression.

## Methods

### Data Sources and Searches

For documentation, we adopted the Preferred Systematic Analyses and Meta-Analysis Reporting Items recommendations (37). A systematic search was conducted from COVID-19 databases from the pandemic inception through October 13^th^, 2021 for full-length articles focusing on the association of increased BMI/Obesity [overweight is defined as a BMI between 25.0 and 29.9, and a BMI of 30 or higher is considered obese (38)] in COVID-19 using a pre-specified data extraction protocol including bibliographic information (year of publication, first author), study information (country, sample size), patient characteristics (age, baseline comorbidities, gender), treatment information and outcome data. The search strategy consisted of keywords “SARS-CoV-2”, “COVID-19”, “Coronavirus”, “Obesity”, “BMI”, “Overweight” “Risk factors” across the three large COVID-19 databases (WHO COVID-19, CDC COVID-19, and LitCOVID PubMed) OVID-Medline Embase, Scopus, Web of Science, and Cochrane Central Controlled Trials Registry. Studies were included from all over the world. There were no language barriers during the literature search. Other literature sources such as the BioRxiv (preprints), MedRxiv (preprints), ChemRxiv (preprints), and SSRN (preprints) were searched as well. We screened the title and abstract of each study identified in the literature search to include eligible articles where obesity or BMI was mentioned as a risk factor and overall comparative results or association with COVID-19 were provided in the abstract. Following this step, we conducted a full text review for final evaluation for study inclusion and data extraction. To discover further eligible studies, we manually searched the reference lists of the included studies, and previously published meta-analysis, systematic review, and the relevant literature. We also scanned the clinicaltrials.gov registry for completed, as well as in-progress randomized controlled trials (RCTs).

### Study Selection

The inclusion criteria for the systematic review are as follows:

1. Studies reporting outcomes such as severity or mortality events of confirmed COVID-19 positive patients, at least one functional endpoint of COVID-19 positive hospitalized patients where body mass index (BMI) values or comparison of obese vs non-obese were provided.

2. Full text and peer-reviewed articles (Case-studies and case series, randomized controlled trials) were included.

**3.** Studies published only in the English language were included.

### Exclusion Criteria

1.Studies published in a language other than English were not considered.

2. Studies with insufficient information were excluded. Case reports, reviews, or nonhuman studies were excluded.

3. Studies focused on patients aged under 18 years old were also excluded.

### Data Extraction and Quality Assessment

The authors (HK and SSR) downloaded all articles from electronic search to EndNote X9 (39) and duplicates were eliminated. Based on the preset eligibility criteria, each study was reviewed by at least two authors (AT, GSS, HK, NJ, YC, RS, SK, KI, and SSR) independently and verified with internal author-reviewer, and disagreements were discussed amongst all author-reviewers and resolved *via* a consensus. The cases included obese Covid-19 positive hospitalized patients and the controls included the non-obese Covid-19 positive hospitalized patients.

**Figure 1:**
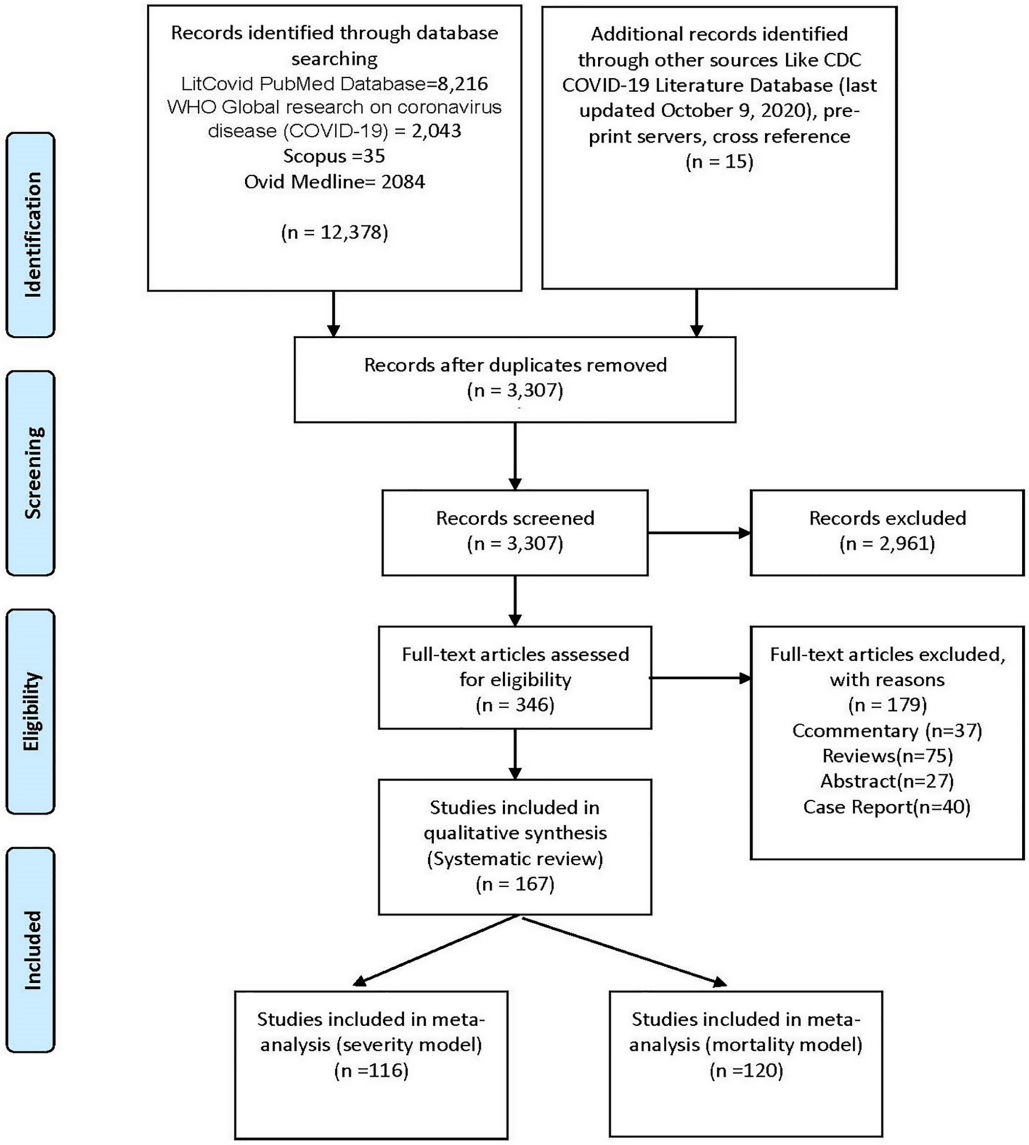
Prisma flow diagram.

Unadjusted and adjusted impact measurements were also extracted where appropriate. From each study, various details including first author name, study type, hospitalized total covid-19 positive patients, the definition of COVID-19 severity, definition of obesity, total obese & non-obese COVID-19 positive patients, patients with high severity and mortality, median age, gender (female sex proportion), proportions of hypertension, pulmonary disease, cardiovascular disease, diabetes, dyslipidemia, liver disease were recorded (**Supplementary Table 1**). The included data was checked for accuracy by all authors. Reporting Items for Systematic Reviews and Meta-Analysis (PRISMA) guidelines were followed (**Figure 1** and **Supplemental Table 2**).

### Risk of Bias Assessment

The NIH study quality assessment Tool was used for measuring the risk of bias in case-control studies and cohort studies (40). The NIH quality assessment tools were based on quality assessment methods, concepts, and other tools developed by researchers in the Agency for Healthcare Research and Quality (AHRQ), Cochrane Collaboration, USPSTF, Scottish Intercollegiate Guidelines Network, and National Health Service Centre for Reviews and Dissemination, consulting epidemiologists and evidence-based medicine experts, with adaptations by methodologists and NHLBI staff (40). Three authors (AT, KI, SA, and SSR) evaluated the likelihood of bias independently, and any conflict was resolved by consensus (**Supplementary Tables 3A–C**).

### Data Synthesis and Analysis

All-cause severity in hospitalized COVID-19 patients with high BMI/obesity was the primary outcome. The severity criteria were defined as the need for ICU admission or the need for mechanical ventilation for the admitted COVID-19 positive patients. If both severities were given in the article, then the category with a higher number of reported events was selected as the severity for COVID-19. The severity rate was evaluated in comparison to the control group (non-obese COVID-19 hospitalized patients). While all-cause mortality in COVID-19 hospitalized patients with high BMI/obesity was the secondary outcome.

The meta-analysis specifically included case-control and cohort studies comparing the effects of high BMI/obesity in COVID-19 hospitalized patients comparing them to the non-obese COVID-19 hospitalized patients. All outcomes were analyzed using the Mantel-Haenszel method for dichotomous data to estimate pooled risk ratio (RR) utilizing the Review Manager (RevMan)- Version 5.4, The Cochrane Collaboration, 2020. Meta-analysis was performed first for studies reporting severity of patients in both groups followed by that for studies reporting severity of disease assuming independence of results for studies that reported both. Due to anticipated heterogeneity, summary statistics were calculated using a random-effects model. This model accounts for variability between studies as well as within studies. Statistical heterogeneity was assessed using Q value and I^2^ statistics.

To explore the differences between studies that might be expected to influence the effect size, we performed random effects (maximum likelihood method) univariate and multivariate meta-regression analyses. The potential sources of variability hypothesized were the gender of the study sample, the proportion of subjects with diabetes, pulmonary disease, dyslipidemia, cardiovascular disease, and hypertension. Covariates were selected for further modeling if they significantly (P < 0.05) modified the association between mortality or severity in the COVID-19 hospitalized patients with high BMI/Obesity. Two models were created, one for severity and the other for mortality of disease as primary and secondary outcomes, respectively. Subsequently, preselected covariates were included in a manual backward and stepwise multiple meta-regression analysis with P = 0.05 as a cutoff point for removal. P < 0.05. (P < 0.10 for heterogeneity) was considered statistically significant. All meta-analysis and meta-regression tests were 2-tailed. The meta-regression was performed with the Comprehensive Meta-Analysis software package (Biostat, Englewood, NJ, USA)14 (41).

We conducted sensitivity analysis with BMI categories (BMI <18 kg/m^2^, BMI 18 kg/m^2^-25 kg/m^2^, BMI 25 kg/m^2^-29.9 kg/m^2^, BMI >30 kg/m^2^, and BMI>40 kg/m^2^) to decrease inherent selection bias in observational studies (42).

## Results

### Study Characteristics of Included Studies

A total of 167 studies, consisting of 3,140,413 COVID-19 patients were included in the meta-analysis. The median age for included patients was 62 (56.4-65.5) with an average of 44.3% female participants (**Supplementary Table 1**). Of the comorbidities considered, 28.1% were diabetics, 22.8% had cardiovascular diseases. For the primary endpoint, i.e. disease severity, a total of 116 studies with predefined severity events with obese vs non-obese were included in the meta-analysis (31, 33, 43–156). These had a combined sample size of 1,685,283 with 117,839 patients reaching the endpoint of high disease severity (**Supplementary Table 1**). Similarly, a total of 120 studies (33, 43–45, 47, 51, 52, 54–59, 61, 62, 64–67, 69, 71, 73–75, 78, 80, 82, 86, 89–91, 93, 96, 99, 100, 104, 106–108, 112–115, 117–120, 122, 124, 125, 127–134, 136, 138, 139, 141–143, 147, 149, 150, 152, 153, 157–207) were included for mortality meta-analysis as a secondary outcome. These had a combined sample size of 1,935,503 with 277,780 patients reaching the endpoint of mortality.

### Meta-Analysis for Severity Outcome

**Figure 2:**
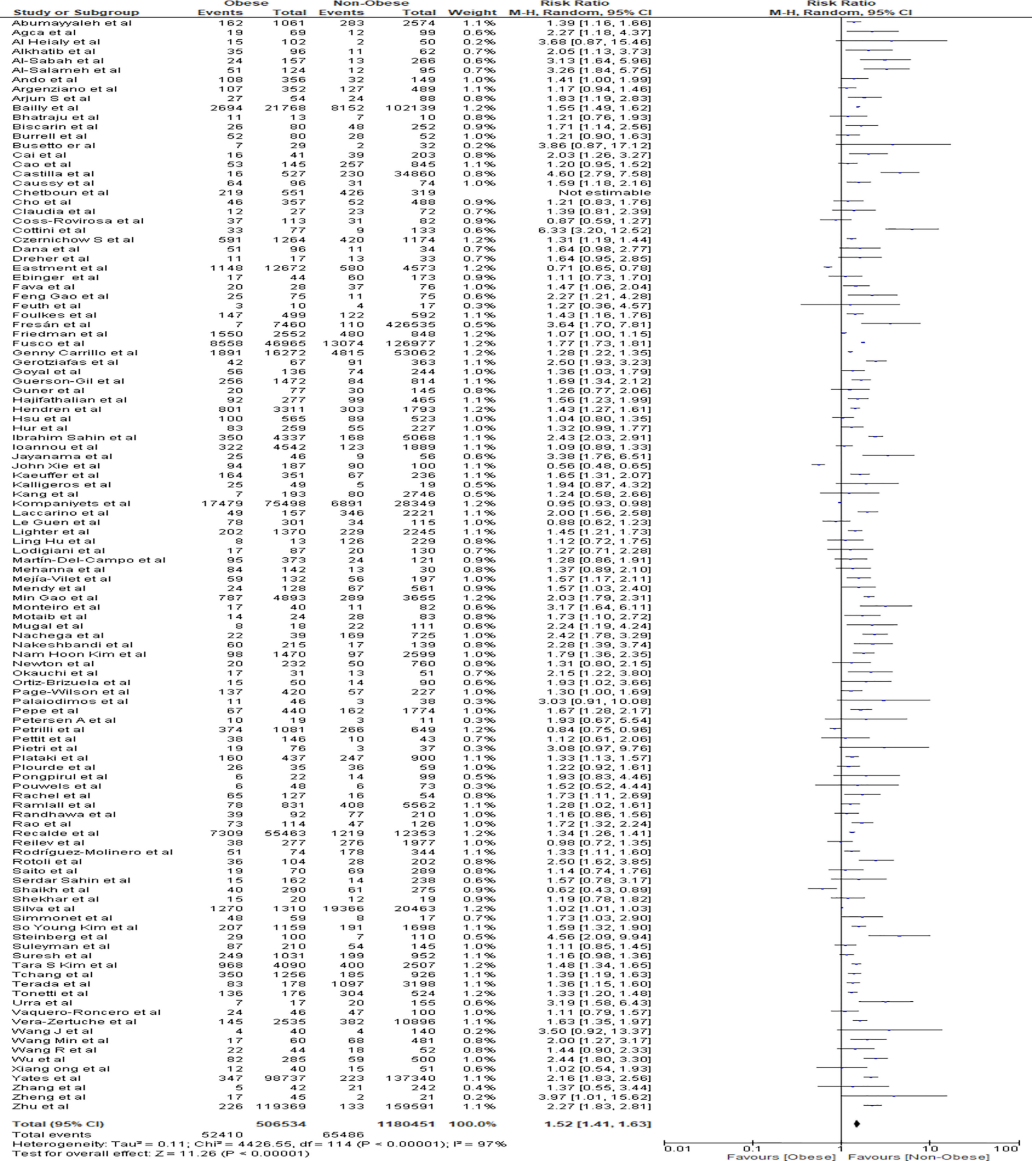
Forest plot for severity analysis.

Findings from the meta-analysis showed that being obese was correlated with increased severity of COVID-19 positive hospitalized patients in comparison to non-obese patients (RR=1.52, 95% CI 1.41-1.63, p<0.001). Heterogeneity was high with I^2^ = 97% (**Figure 2**).

### Meta-Analysis for Mortality Outcome

**Figure 3:**
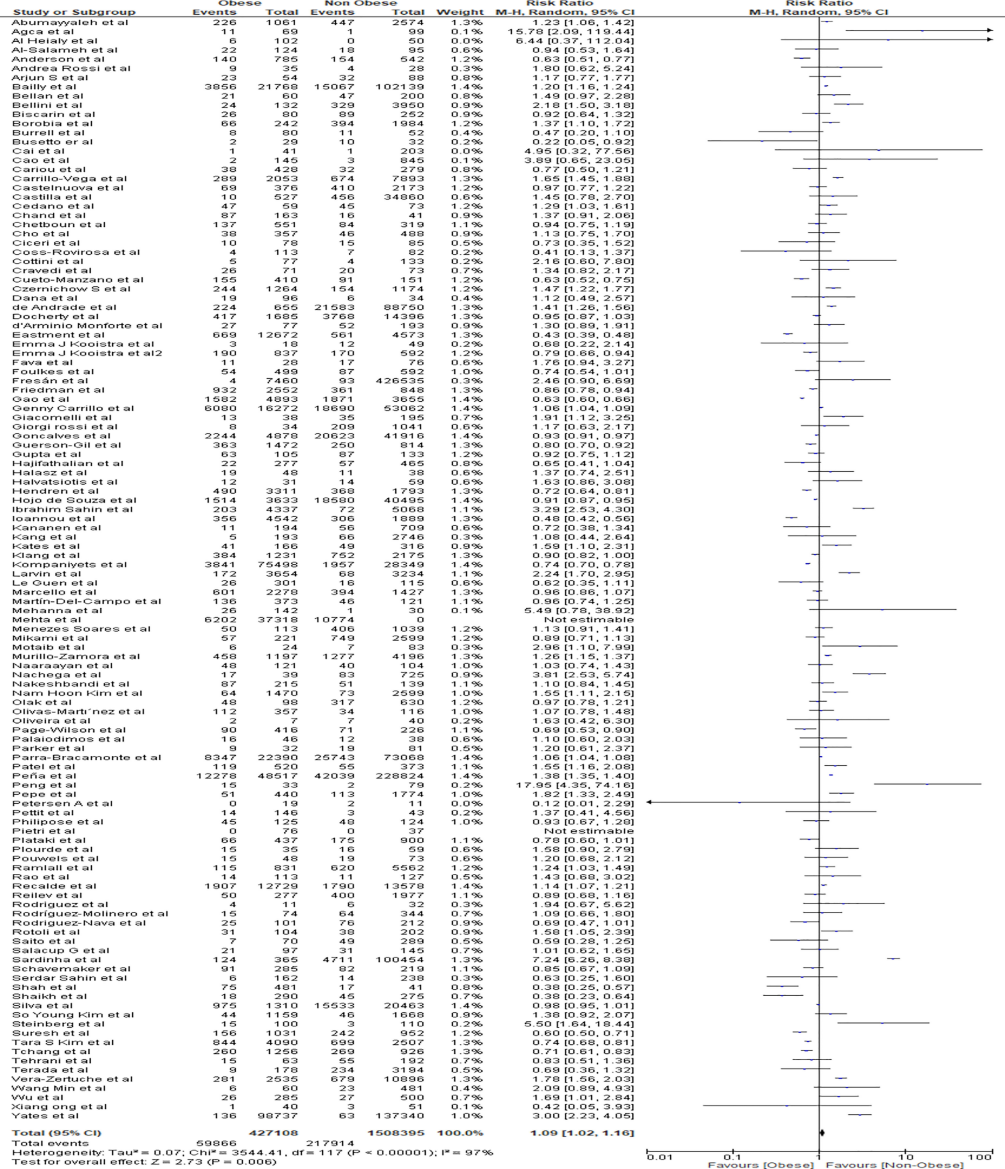
Forest plot for mortality analysis.

Meta-analysis findings showed that obesity was associated with an increased risk of mortality in obese patients from COVID 19 infections in comparison to the non-obese patient population (RR=1.09, 95% CI 1.02-1.16, p=0.006). Heterogeneity was high with I^2^ = 97% (**Figure 3**).

### Multivariate Meta-Regression Model for Severity Outcome

**Figure 4:**
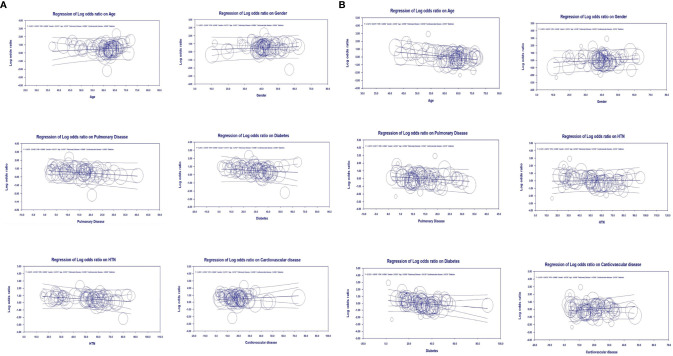
Meta-regression analyses, **(A)** Severity meta-regression analysis, **(B)** Mortality meta-regression analysis.

Multivariate meta-regression was performed to explain variations in the association between COVID-19 severity and obesity. We found that age, female gender, the proportion of pulmonary disease, diabetes, cardiovascular diseases, and hypertension covariates to be significant, and this explained R^2^ = 40% of the between-study heterogeneity in severity. **Figure 4A** shows the resulting equation and individual covariate effect graphs.

### Multivariate Meta-Regression Model for Mortality Outcome

Multivariate meta-regression was performed to explain variations in the association between mortality and obesity, and revealed that age, female gender, the proportion of pulmonary disease, diabetes, hypertension, and cardiovascular diseases were significant together. Overall, these covariates together explained R^2^ = 50% of the between-study heterogeneity in mortality. **Figure 4B** shows the resulting equation and individual covariate effect graphs.

### Sensitivity Analysis

We did not find any statistical significance for risk of mortality with COVID-19 when analyzed by BMI categories during sensitivity analysis (**Supplementary Figures 1A–C**). However, we observed that underweight status (BMI<18 kg/m^2^) is associated with increased risk of mortality in COVID-19 (RR 1.50, 95% CI 1.36-1.65, p=<0.001; I^2^ = 46%) (**Supplementary Figure 1D**) but not statistically significant to severity of COVID-19 (RR 1.04, 95% CI 0.85-1.28, p=0.69; I^2^ = 83%) (**Supplementary Figure 1E**) as compared to normal BMI category of 18-24.99 kg/m^2^. Normal weight is protective to COVID-19 disease severity compare to overweight (BMI 25-29.9 kg/m^2^) (RR 0.75, 95% CI 0.69-0.82, p=<0.001; I^2^ = 88%), Class 1 and Class 2 obesity (BMI of 30-39.99 kg/m^2^) (RR 0.67, 95% CI 0.60-0.74, p=<0.001; I^2^ = 94%) and Class 3 obesity (BMI >40 kg/m^2^) (RR 0.77, 95% CI 0.68-0.88, p=<0.001; I^2^ = 89%).

### Publication Bias

Visual inspection of the standard error plots for the severity analysis also (**Supplementary Figure 2A**) suggests symmetry without an underrepresentation of studies of any precision. However, in Egger’s regression test the null hypothesis of no small study effects was rejected at p<0.05 (estimated bias coefficient = -0.27 ± 0.16SE).

Similarly, visual inspection of the standard error plots for the mortality analysis (**Supplementary Figure 2B**) suggests symmetry without an underrepresentation of studies of any precision. Corroborating inspection findings, Egger’s regression test, the null hypothesis of no small study effects, was rejected at p<0.05 (estimated bias coefficient = -0.20 ± 0.15SE).

## Discussion

### Summary of Result

In our study, we found that obesity has a strong association with increased severity and mortality of COVID-19 infection. Our results suggest that obese individuals are 1.5 times more likely to experience severe outcomes and 1.09 times more likely to die when compared to non-obese individuals with COVID-19 disease. Our meta-regression severity model suggested that 40% of the heterogeneity could be explained by age, gender, diabetes, hypertension, pulmonary and cardiovascular diseases. The mortality meta-regression model suggested that 50% of the heterogeneity could be explained by age, gender, diabetes, hypertension, pulmonary and cardiovascular diseases. Through these regression models, we were able to address the major amount of heterogeneity seen in our meta-analysis.

### Comparison With Existing Literature

Various meta-analyses were conducted to evaluate the association of obesity with mortality and severity in critically ill patients (208–210). The results were not universal, despite a wide variety of observations. In a total of 62,045 critically ill patients, Akinnusi et al. compared the ICU mortality between obese and non-obese patients and found no dissimilarities (208). Hogue et al. (n=22) conducted a meta-analysis of 88,051 patients and found that obesity did not impact ICU mortality (209). However, Oliveros and Villamor et al. found that ICU mortality was increased only in underweight patients and reduced in overweight and obese patients (210). In another study, Zhao et al. observed that having a high BMI is related to a longer duration on mechanical ventilation but lower mortality (211). We also found four meta-analyses (studies n=6, 17, 40, 76) (212–215) that explored the association of obesity and worse outcomes in COVID-19 and found a similar association. On the contrary, one study (216) refuted the possibility of this association. Owing to their small sample population (Studies n=2), it is likely that they were underpowered to tease out the true difference or association (216). With a much larger sample size (n=167) our study provides more robust evidence to establish this association.

Over the last year, five meta-regression studies evaluating the direct relationship between obesity and COVID-19 have been published. Yang et al. (studies n=41) concluded that in COVID-19 patients obesity is associated with increased mortality, increased rates of hospitalization, ICU admissions, and the need for mechanical ventilation. However, they found no confounding factors causing heterogeneity regarding hospitalization, ICU admission, and in-hospital mortality of COVID-19 patients (217). In another such study, Mesas et al. (studies n=60) described that obesity was linked to increased mortality only in studies with fewer chronic or critical patients and reported the mean age of patients as the most important source of heterogeneity, followed by sex and health condition (218). Soereto et al. (studies n=16) reported that patients with higher BMI were at increased risk of developing ‘poor outcomes’ - defined as mortality, ICU admission, ARDS incidence, severe COVID-19, need for mechanical ventilation, and hospitalization. In their meta-regression, the heterogeneity in poor outcomes was explained by age, type 2 diabetes mellitus, hypertension, and gender (219). Cai Z. et al (220) also published meta-analysis results involving 46 studies and a population size of 625,153 patients. They also found similar results as our meta-analysis, wherein patients with obesity have a higher risk of hospitalization, ICU admission, and mechanical ventilation. We have improved upon that and analyzed 167 studies involving more than 3.14 million patients and achieved similar results. Another meta-analysis and regression study by Poly TN et al (213) included 17 studies and reiterated that patients with class III obesity are at more risk than patients with class II or class I obesity. Du et al (148) and Chu et al (149) (studies n=16 and 22, respectively) found that the association between obesity and COVID-19 severity and mortality was significantly influenced by age, but not by gender or other comorbidities. Our meta-regression identified the likely confounders to be age, gender, and co-morbidities. Through this model, we were able to explain high heterogeneity with the highest number of confounders, which other meta-regressions in recent literature were not able to reach and many did not define high heterogeneity in their analysis (217–219, 221, 222). Thus, we established the remarkable, strong association that obesity plays in worsening these outcomes in patients with COVID-19 infection.

In the sensitivity analyses, we were only able to find statistically significant results for increased mortality in BMI<18 kg/m^2^ as compared to BMI 18 kg/m^2^-25 kg/m^2^, however, such significance was not noted in any other BMI categories with severity and mortality in COVID-19. This could be due to BMI being a crude estimate of adiposity, and that it may not be sensitive enough to tease out the real differences. However, in their study, Anderson et al (157) found that patients with obesity have a greater chance of intubation or mortality, with people with class 3 obesity having the greatest risk compared to overweight patients.

### Pathophysiological Connection of Obesity and COVID-19 Infection

Obesity is known to be associated with many comorbid conditions (223), including hypertension, atherogenic dyslipidemia, cardiovascular disease, insulin resistance or type 2 diabetes, and altered cortisol metabolism, etc (224). Various biological mechanisms contribute towards increased risk of severity or mortality upon COVID-19 infection in obese patients. First, ectopic fat exacerbates the inflammation caused by COVID-19 by the upregulation of proinflammatory cytokines like interleukin-6 (IL-6) and tumor necrosis factor-alpha (TNF-α), angiotensin II (ATII), and prothrombotics (225–227). Second, obese patients are found to have decreased levels of anti-inflammatory adipokine, adiponectin, which is linked to an increased level of ATII (228, 229). Obesity is associated with overexpression of ACE2 receptors which may aid infection and serve as viral reservoir (230). Further, coronavirus reduces the activity of ACE2 inhibitors, which again leads to an increase in the ATII level (231, 232). Higher levels of ATII lead to progression of lung injury among COVID-19 patients by triggering the NADH/NADPH oxidase system and promoting fibrosis, contraction, and vasoconstriction (233, 234). Moreover, it is associated with endothelial dysfunction (235), the key pathogenic event in COVID-19 leading to mortality and morbidity (236, 237). Furthermore, an increased expression of inflammatory adipokine molecules enhances the production of cytokines TNF-α and IL-6, which are associated with alveolar damage that leads to higher severity and mortality (238). Obesity or increased adiposity plays a key role in endothelial dysfunction by activating several cascades of pathological events, namely activation of renin-angiotensin system (239), activation of procoagulant/hypercoagulation pathway (240), activation of proinflammatory mediators (241), insulin resistance (242), oxidative stress (243), platelet dysfunction (244), and immune dysregulation (245). In the study by Danzinger et al (246) obesity was found to be associated with increased incidence of acute kidney injury and an increase in short- and long-term mortality. These events are summarized in **Supplementary Figure 3**.

### Public Health Implication

The COVID-19 pandemic has created a multitude of concerns globally, and public health providers are working towards minimizing the damaging effects of COVID-19. There is no direct and effective treatment available to control the infection, thus, global morbidity and mortality increase day by day (5). COVID19 shows a wide spectrum of symptoms; many individuals recover without many health complications. However, some infected patients had severe symptoms which required hospitalization, care in intensive care units (ICU), prolonged symptom management, still many succumbed to death (247). Elderly patients were more vulnerable to severe outcomes because they have had multiple diseases and associated risks. A significant number of studies reported that elderly patients and patients with diabetes, stroke, CKD, and COPD are associated with poor outcomes (248, 249). Obesity, especially, class 3 obesity, was associated with an increased rate of mortality among patients infected with COVID-19. Similarly, during the previous H1N1 pandemic, patients with obesity observed prolonged hospitalization, mechanical ventilation, and increased mortality when it was calculated as an independent risk factor (250, 251).

Several population-based cohort studies reported that obesity is linked to increased comorbidities like diabetes, hypertension, and heart disease. Importantly, the mortality rate among patients with obesity proportionally increased with BMI (22, 252). Moreover, obesity makes patients’ conditions worse if patients develop infections by downregulating the inflammatory cascade. Hyperactivation of inflammatory pathways alter the level of cytokines, adiponectin, and leptin and distort both macro- and micro-vascular responses (22, 252, 253). Obesity is also associated with lung function impairment, which involves altering mechanics and airway resistance and decreasing the gas exchange (254, 255). The findings of our study suggest that health care providers and physicians should pay attention to the obesity status of COVID-19 patients because this group of patients is at high risk of worse consequences. The conclusions of our study as well as of others, highlight the need for vigilance, and an earlier start to treatment in obese patients with COVID-19 infection (256, 257) as obese patients had higher hospitalization, ICU care, a requirement of mechanical ventilation with poor prognosis, and worse outcomes.

### Effect of COVID-19 on Obesity and Prevention/Treatment Strategies for Patients With Obesity

COVID-19 plays a role in the emergence of obesity in this regard. The public health response to the COVID-19 pandemic is mostly based on restricting human contact and isolation, which affects people’s behavior, and is linked to an increased risk of mental disease (258) and adds to increased incidence of obesity (259). Maintaining a healthy body weight requires regular physical activity, which was cut down during the isolation required during the COVID-19 pandemic (260). People tend to overeat as a result of increased anxiety and monotony, resulting in the consumption of additional energy/calories and an intense desire for food (261). Similarly, quarantine during the COVID-19 outbreak has led to an economic burden, and in some cases, this might mean people having to choose cheaper, less healthy meals. These foods are processed and associated with more fat, carbohydrates, and higher calorie intake (262), which is more likely to cause weight gain than a balanced healthy diet (263).

Obesity must be avoided at all costs. Increased physical activity and calorie restriction are typically used to lose weight. For weight maintenance, it is recommended that people exercise for more than 300 minutes each week (264). People use a range of weight-loss tactics to achieve this, such as consuming fewer calories, daily exercise, intermittent fasting, and using weight-loss medications or diuretics (265). Decreasing calorie consumption is by far the most popular method for weight reduction (266, 267). Metformin usage was reported to be strongly linked with a decrease in COVID-19 mortality in one study (268). This discovery might be explained by a number of factors. Metformin usage was reported to be strongly linked with a decrease in COVID-19 mortality in another recent study (269). This discovery might be explained by a number of factors (268). First, metformin inhibits SARS-CoV-2 from attaching to the receptor (270). Second, metformin suppresses SARS-CoV-2 infectivity and COVID-19 mortality by inhibiting the mTOR signaling pathway (268). Finally, metformin has been shown to reduce inflammatory responses (271). Metformin also lowers the risk of negative outcomes in COVID-19 individuals by lowering their BMI and body weight (272).

### Strengths and Limitations

The prime strength of this study is the large sample size. With an exhaustive search strategy, we compiled 167 studies conducted globally. We also added the most recent studies to our meta-analysis and meta-regression model including those that reported contradictory information. This enabled us to arrive at a more definitive conclusion about the risk associations. To define the heterogeneity in the meta-analysis, we also conducted a meta-regression analysis. For moderators, we used the most probable confounders based on the available evidence. This enabled us to delineate the impact of obesity as an independent risk factor for mortality and severity in COVID-19.

We included five studies from preprint databases (78, 102, 109, 146, 200) that may not be comparable to peer-reviewed articles in terms of their quality of methodology. However, given the time-sensitive nature of this pandemic, the benefit of early dissemination of critical information and its inclusion in various analyses outweighs the risk from minor methodological flaws. The second factor was the heterogeneity in the studies in terms of the study design and methodology, patient sample, and treatment received. There was a lack of uniformity in the type of outcomes evaluated for severity and their definitions in different studies. For the same reason, it was not possible to deduce the effect of obesity on individual outcomes. The third limitation is that the analysis was undertaken for hospitalized patients only, meaning we cannot generalize our results for patients treated in outpatient clinics or at home. Analyzing outpatient data may help us gain a complete picture of the impact of obesity on overall COVID-19 outcomes. The fourth limitation is that our analysis did not compare the outcomes with respect to visceral obesity and only BMI was used. However, it was beyond the scope of this analysis because of the lack of those details in most included studies. We suggest that prospective studies should obtain and report this information about their sample population. Lastly, it is possible that some confounders, which could have otherwise accounted for the residual heterogeneity, were not evaluated in the meta-regression analysis due to limited information.

### Conclusion

Our findings suggest that obesity significantly increases the risk of severity and mortality in hospitalized COVID-19 patients. Therefore, the inclusion of obesity or surrogate body mass index in prognostic scores and streamlining the management strategy and treatment guidelines to account for the impact of obesity would be vital to improving patient outcomes in hospitalized COVID-19 patients. Our findings also serve as a call for the scientific community to delve further into its pathophysiology and identify potential pharmacological targets, since COVID-19 is an ever-evolving disease. Finally, this information must be disseminated to the general public to intensify the primary prevention of obesity.

## Data Availability Statement

The datasets presented in this study can be found in online repositories. The names of the repository/repositories and accession number(s) can be found in the article/**Supplementary Material**.

## Author Contributions

Authors RS and SSR contributed equally to defining the study outline and manuscript writing and are co-primary authors. Data review and collection were performed by AT, GSS, HK, KI, NJ, RS, SK, AP, YC, and SSR; statistical analysis was undertaken by AB, SK, and VB; risk of bias was done by AT, SA, KI, NS, and SSR. Study design and the distribution of articles for critical review were performed by IM, VP, RK, and VB. All authors approved the final version of the published study. RS, SSR, VB, and VP are the guarantors of the published work, and take responsibility for the integrity of the work as a whole, from inception to the published article. All authors contributed to the article and approved the submitted version.

## Conflict of Interest

The authors declare that the research was conducted in the absence of any commercial or financial relationships that could be construed as a potential conflict of interest.

## Publisher’s Note

All claims expressed in this article are solely those of the authors and do not necessarily represent those of their affiliated organizations, or those of the publisher, the editors and the reviewers. Any product that may be evaluated in this article, or claim that may be made by its manufacturer, is not guaranteed or endorsed by the publisher.
