# Supplementary material for: Association of Obesity With COVID-19 Severity and Mortality: An Updated Systemic Review, Meta-Analysis, and Meta-Regression
Source: Front Endocrinol (Lausanne). 2022 Jun 3;13:780872. doi: 10.3389/fendo.2022.780872 (PMC9205425; doi:10.3389/fendo.2022.780872)
Supplement: Supplementary file 1 [file DataSheet_1.docx]

**Supplementary Figure 1: Sensitivity analysis for Mortality by BMI categories**

**Supplementary Figure 1a: Sensitivity analysis for mortality for BMI 18-24.99 kg/m^2^ vs BMI 30-39.99 kg/m^2^**  **
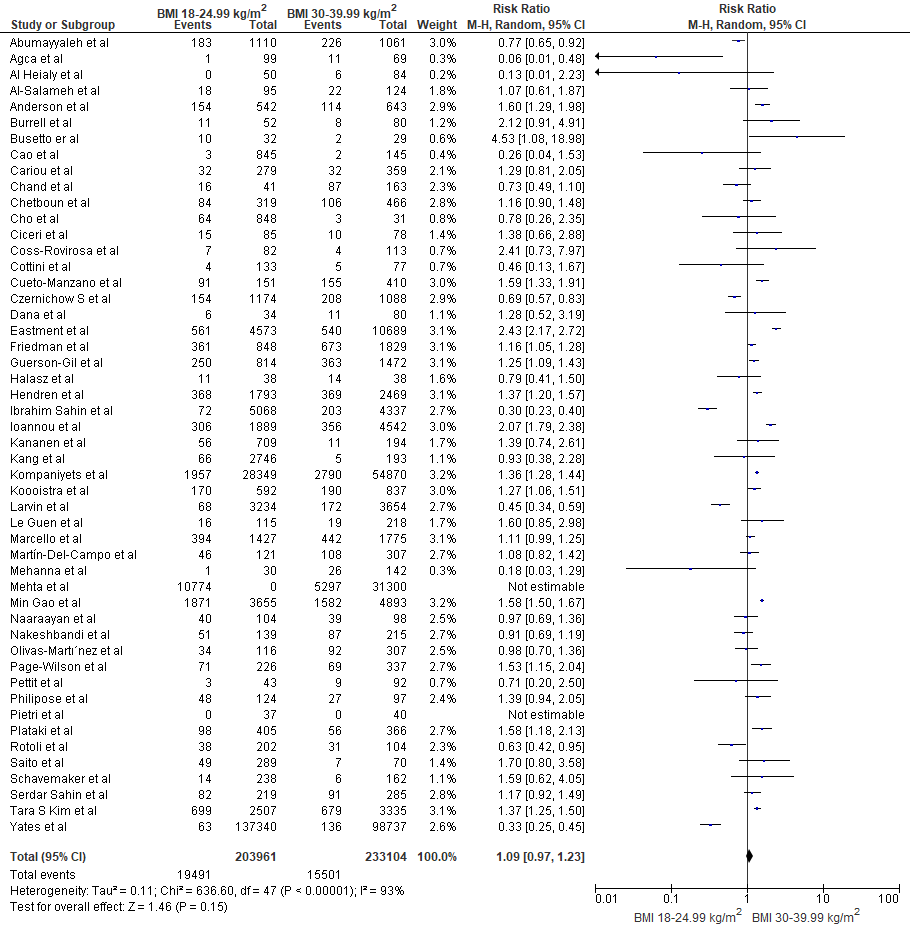
**

**Supplementary Figure**
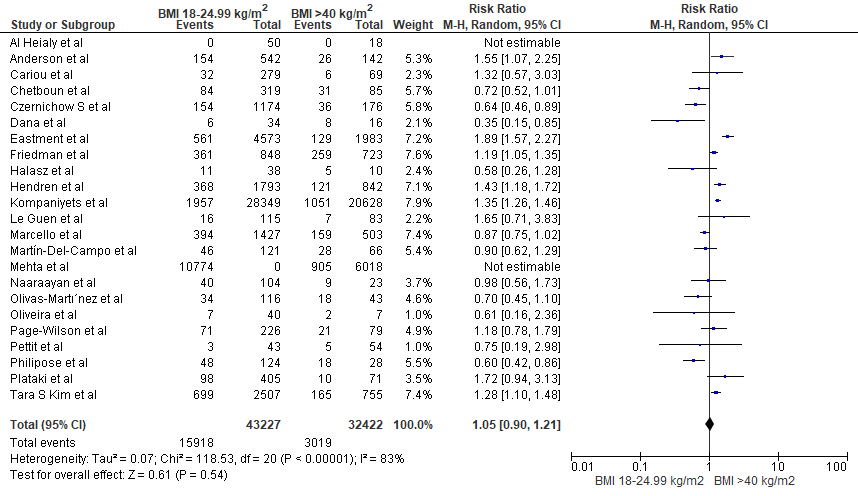
**1b: Sensitivity analysis for mortality for BMI 18-24.99 kg/m^2^ vs BMI >40 kg/m^2^**

**
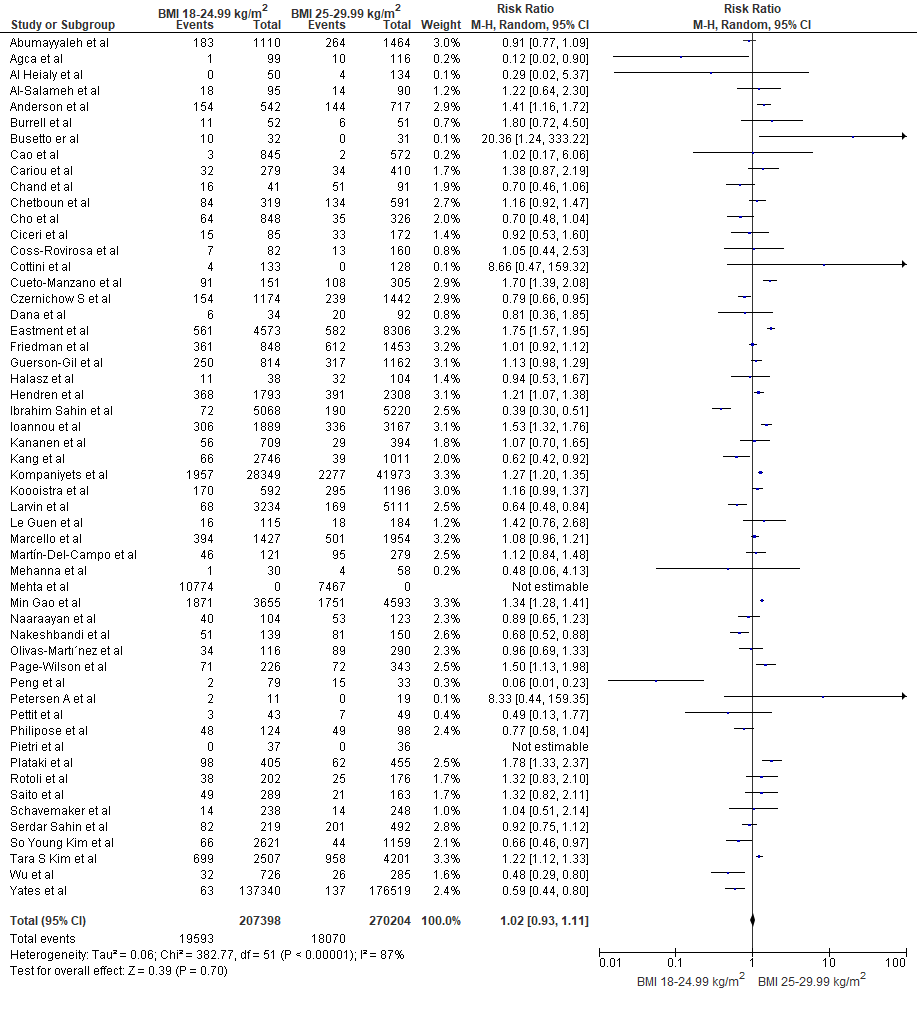
 Supplementary Figure 1c: Sensitivity analysis for mortality for BMI 18-24.99 kg/m^2^ vs BMI 25-29.99 kg/m^2^**

**Supplementary Figure 1d: Sensitivity analysis for mortality for BMI <18 kg/m^2^ vs BMI 18-24.99 kg/m^2^**

**
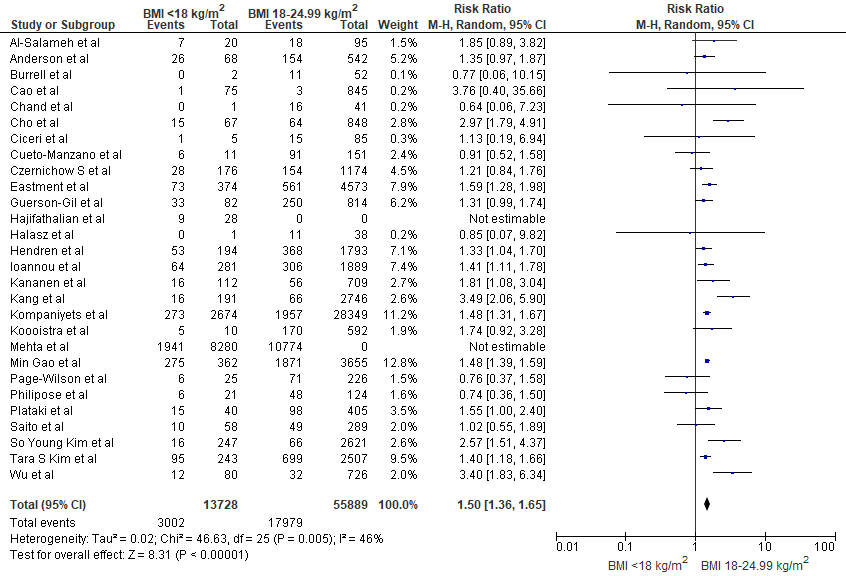
**


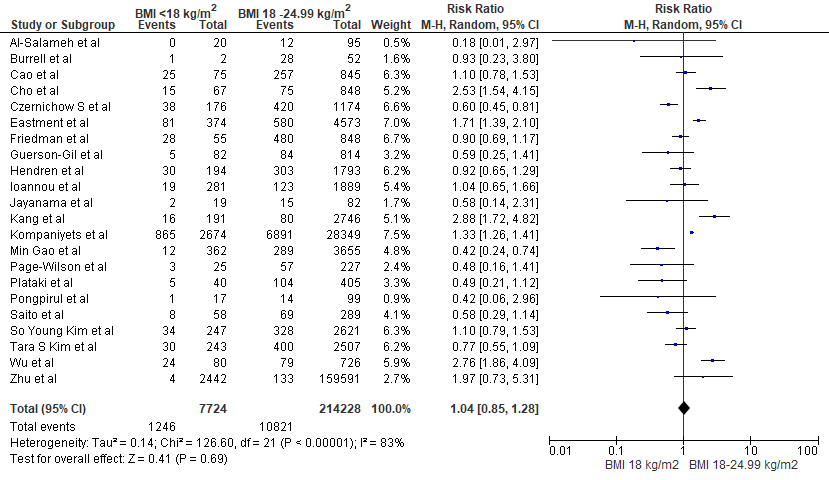
 **Supplementary Figure 1e: Sensitivity analysis for severity of COVID-19 BMI <18 kg/m^2^ vs BMI 18-24.99 kg/m^2^**


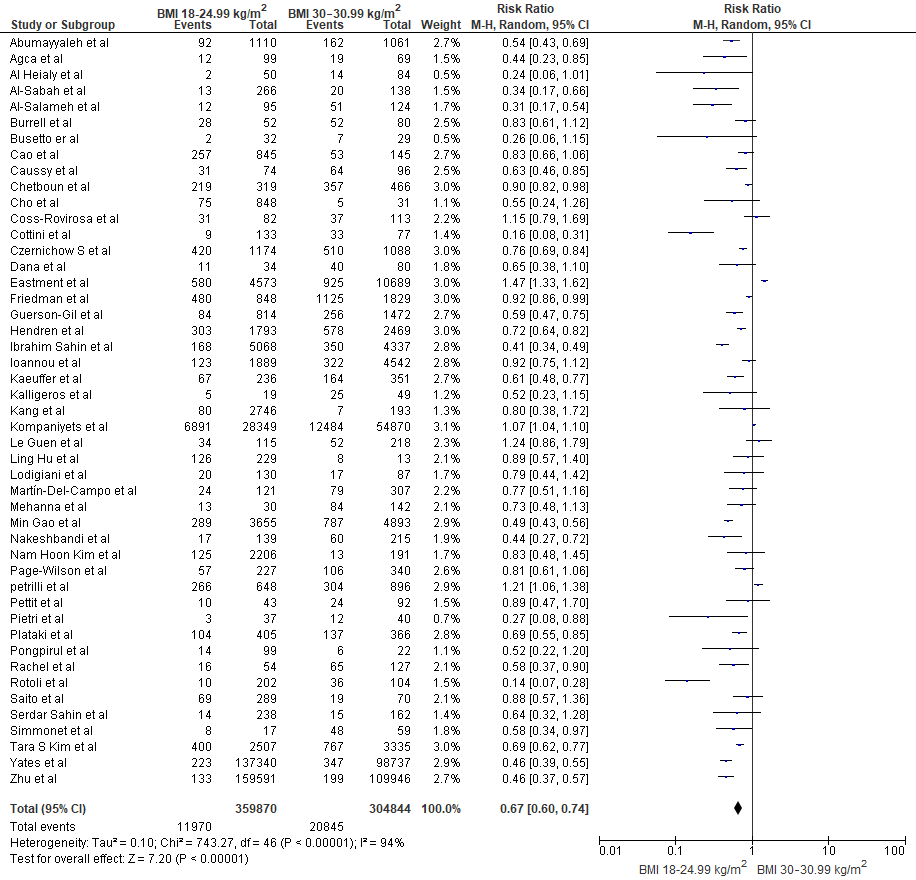
 **Supplementary Figure 1f: Sensitivity analysis for severity of COVID-19 BMI** **18-24.99 kg/m^2^ vs BMI 30–39.99 kg/m^2^**

**Supplementary Figure 1g: Sensitivity analysis for severity of COVID-19 BMI 18-24.99 kg/m^2^ vs
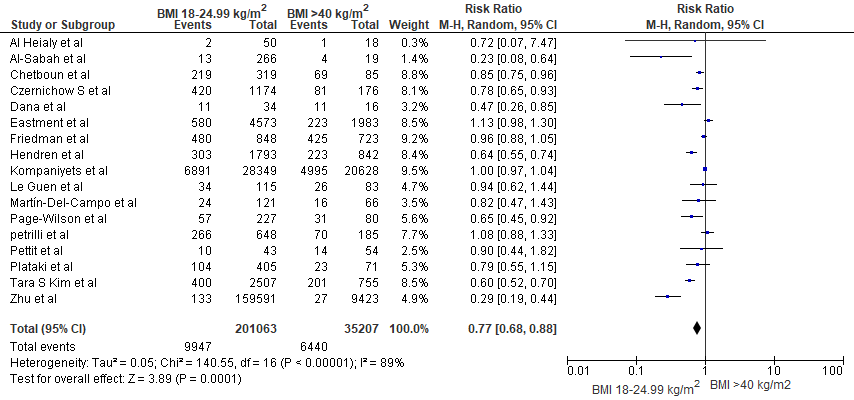
BMI** **>40 kg/m^2^**


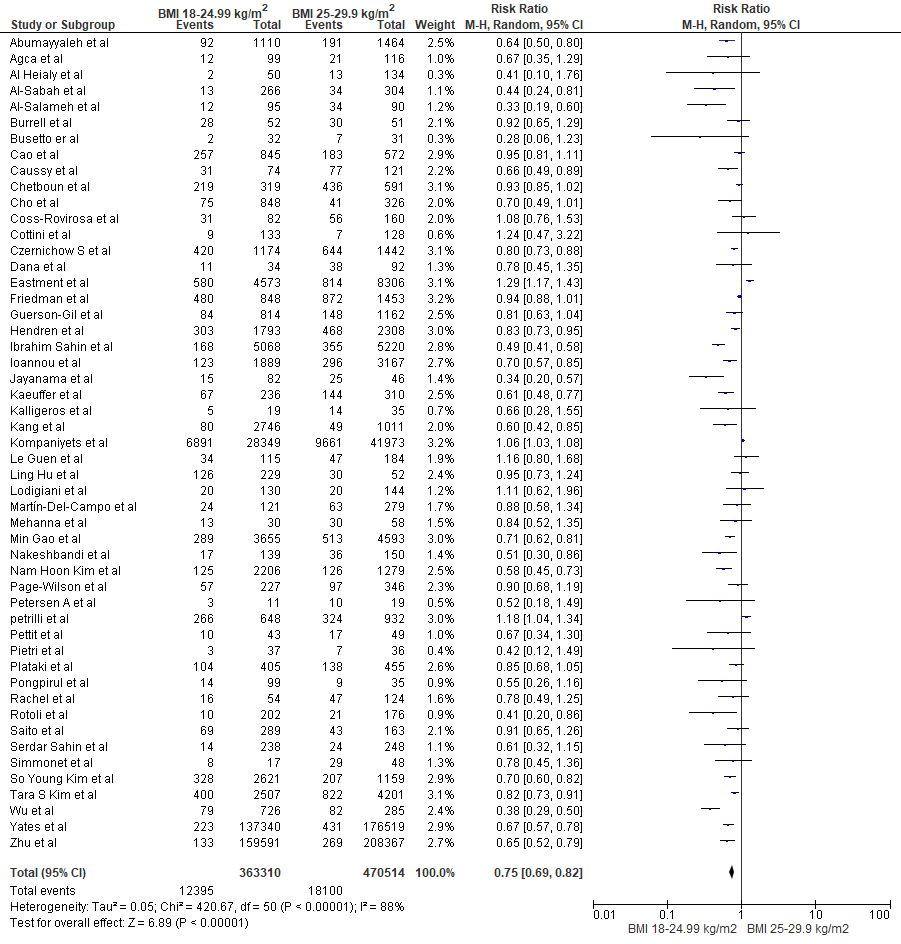
 **Supplementary Figure 1h: Sensitivity analysis for severity of COVID-19 BMI 18-24.99 kg/m^2^ vs** **BMI 25-29.9 kg/m^2^**

**Supplementary Figure 2: Standard error funnel Plot (Publication bias)**

**Supplementary Figure 2a:** **Standard error plot for severity analysis**

| **Egger Regression** | | | |  |
| --- | --- | --- | --- | --- |
|  | Estimate | SE | CI LL | CI UL |
| Intercept | 1.64 | 0.39 | 0.87 | 2.41 |
| Slope | -0.27 | 0.16 | -0.59 | 0.05 |
|  |  |  |  |  |
| t test | 4.22 |  |  |  |
| p-value | 0.000 |  |  |  |

**Supplementary Figure 2b:** **Standard error plot for mortality analysis**

| **Egger Regression** | | | |  |
| --- | --- | --- | --- | --- |
|  | Estimate | SE | CI LL | CI UL |
| Intercept | 0.76 | 0.40 | -0.03 | 1.55 |
| Slope | -0.20 | 0.15 | -0.50 | 0.11 |
|  |  |  |  |  |
| t test | 1.90 |  |  |  |
| p-value | 0.060 |  |  |  |


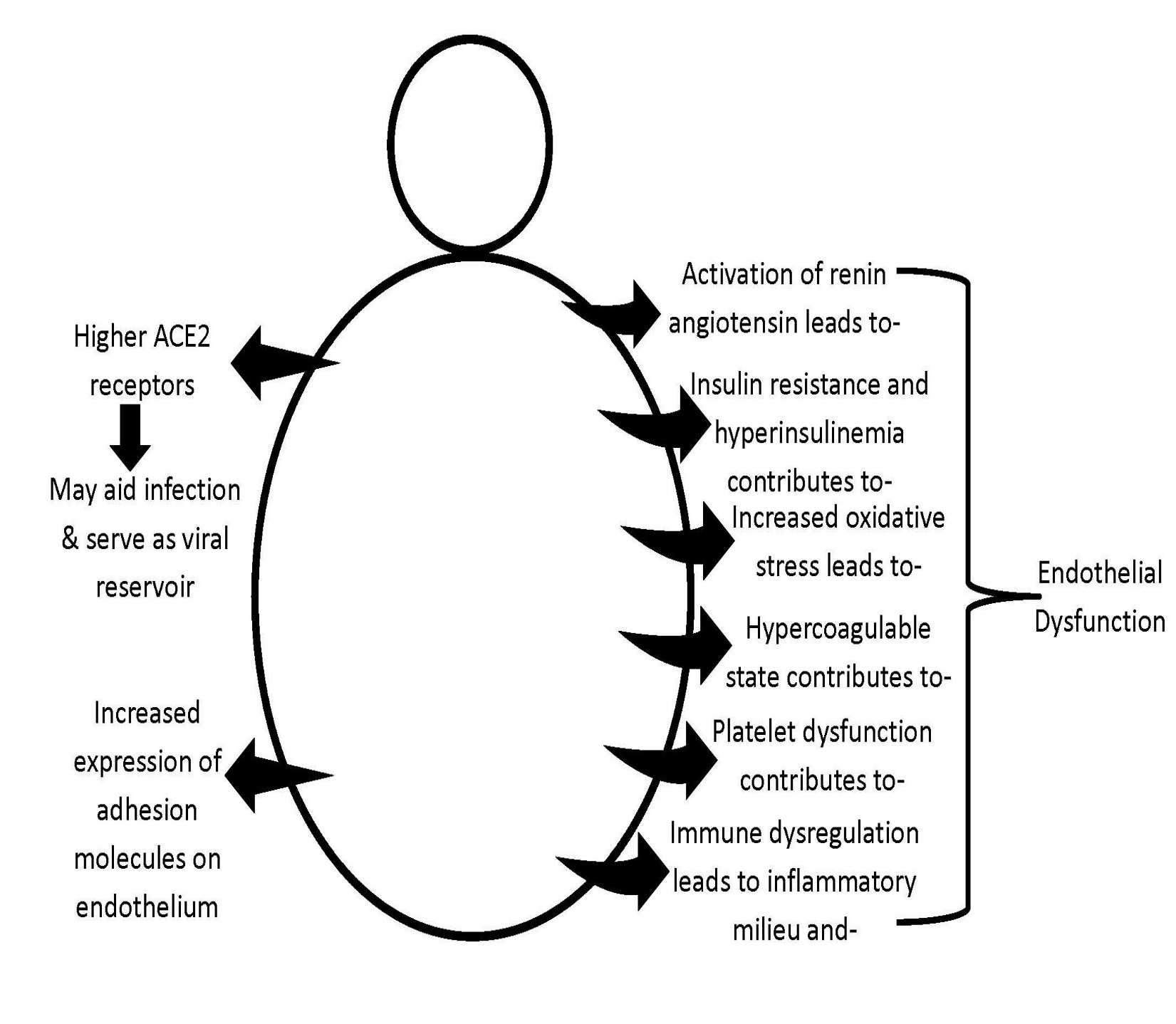
 **Supplementary Figure 3:** **Several mechanisms of obesity’s role in endothelial dysfunction: A central event in pathogenicity of COVID-19 infection**
