# Supplementary material for: Association of Obesity With COVID-19 Severity and Mortality: An Updated Systemic Review, Meta-Analysis, and Meta-Regression
Source: Front Endocrinol (Lausanne). 2022 Jun 3;13:780872. doi: 10.3389/fendo.2022.780872 (PMC9205425; doi:10.3389/fendo.2022.780872)
Supplement: Supplementary file 2 [file Table_1.pdf]

Supplementary Table 1: Baseline characteristics of the included studies.

Reference citation number so for each study correspond to the reference list in the main article

STUDY	COUNTRY OF STUDY	STUDY DESIGN	DEFINITION OF SEVERITY	TOTAL COVID-19 POSITIVE PATIENTS	TOTAL PATEINTS WITH OBESITY	TOTAL PATEINTS WITH NORMAL WEIGHT	MEDIAN AGE	FEMALE SEX (%)	HYPERTENSION (%)	PULMONARY DISEASE (%)	CARDIO VASCULAR DISEASE (%)	DIABETES (%)
**Abumayyaleh et al[43]**	multi-country	Retrospective cohort	IMV	3635	1061	2574	63	41.8	50.3	22.5	23.2	19.2
**Agca et al[44]**	Turkey	Retrospective Cross-sectional Study	ICU admission	284	69	99	54	43	31	19	11	2
**Al Heialy et al[45]**	United Arab Emirates	Cohort	ICU admission	286	102	50	46.9	27.2	25.8	3.8	3.8	26.2
**Al-Sabah et al[46]**	Kuwait	Cohort	ICU admission	1158	157	266	40.5	18.4	20.4			23.4
**Al-Salameh et al[47]**	France	Retrospective	ICU admission	329	124	95	72	43.5	61.4	10.3	35	28.3
**Alkhatib et al[48]**	United States	Retrospective cohort	ICU admission	158	96	62	57	61.4	67.7	17.5	13.3	48.1
**Anderson et al[157]**	United States	Retrospective cohort	Not valid	2466	785	542	67	42	52	17	7	40
**Ando et al[49]**	USA	Cohort	Critical care	1075	356	149	65.5 ± 15.2*	47.5				15
**Andrea Rossi et al[158]**	Italy	Cohort	Not valid	95	35	28		18	47.4		38.9	19
**Argenziano et al[50]**	United States	Cohort	ICU admission	1000	352	489	63	40.4	60.1	22.3	23.3	37.2
**Arjun S et al[51]**	United States	Cohort	ICU admission	142	54	88						
**Bailly et al[52]**	France	Retrospective cohort	IMV	134209	21768	102139	63.8 ± 19.8	47.6	49.6	16.9	21.9	24
**Bellan et al[159]**	Italy	Cohort	Not valid	407	60	200	71	41	58	3	30	24
**Bellini et al[160]**	Italy	Observational Study	Not valid	4481	156	3950		49.5	5.6	9.7	29.5	10.3
**Bhatraju et al[53]**	United States	Case series	ICU admission	24	13	10	64	38		16		58

**Biscarini et al**[54]	Italy	Retrospective cohort	ICU admission	427	80	252	67	31.8	50		28	19
**Borobia et al**[161]	Spain	Cohort	Not valid	2226	242	1984	61	51.8	41.3	13.3	19.3	17.1
**Burrell et al.**[55]	Australia	Cohort	Mechanical ventilation	204	80	52	63.5	31	24	11	20	28
**Busetto er al**[56]	Italy	Cohort	ICU admission	92	29	32	70.5	39.1	64.1	13	31.5	30.4
**Cai et al**[57]	China	Cohort	ICU admission	383	41	203	48	52.2	15.1		4.9	7.3
**Cao et al**[58]	China	Retrospective observational	Severe pneumonia	1637	145	845		50.3	28.9	2.8	5.5	14.1
**Cariou et al**[162]	France	Cohort	Mechanical ventilation	1117	428	279	69.8	35.1	77.2	10.4	11.6	88.5
**Carrillo-Vega et al**[163]	Mexico	Cohort	Not valid	3922	987	7893	54.2	35	34.1	8.1	5.1	30
**Castelnuova et al**[164]	Italy	Cohort	Not valid	3894	376	2173	67	38.3	49.4	14.3	21.1	19
**Castilla et al**[59]	Spain	Prospective cohort	ICU admission	35387	527	34860		51.5	12.8	6.6	7.7	5.3
**Caussy et al.**[60]	France	Cohort	Mechanical ventilation	291	96	74						
**Cedano et al**[165]	United States	Cohort	Not valid	132	59	73	63	41	59	13	24	45
**Chand et al**[166]	United States	Cohort	Not valid	300	163	41	58.2	39.3	66.7	18.7	13.7	44.7
**Chetboun et al**[61]	Europe, Israel, USA	Retrospective cohort	IMV	1461	551	319	64	26.8	51.5	10	25.5	29.2
**Cho et al**[62]	South Korea	Retrospective cohort	IMV/multiorgan failure/ECMO/death.	1272	357	488		59	44.8	4.8	10	26.1
**Ciceri et al**[167]	Italy	Cohort	Not valid	410	78	85	65	27.1	49.5	5.3	12.4	17.8
**Claudia Gregoriano et al**[63]	Switzerland	Cohort	ICU admission	99	27	72	67	37	57	21	28	22
**Coss-Rovirosa et al**[64]	Mexico	Retrospective cohort	ICU admission	355	113	82		33.8	27.9			17.2
**Cottini et al**[65]	Italy	Prospective Cohort	ICU admission with intubation	338	77	133	65.7	41.6	45.2	10.6	24.9	24.3

**Cravedi et al[168]**	United States, Italy, and Spain	Retrospective cohort	Not valid	144	71	73	62	34.02	95	18.8	28	52
**Cueto-Manzano et al[169]**	Mexico	Prospective Cohort Study	Not valid	1010	410	151	58	37	54	9	8	40
**Czernichow, S et al[66]**	France	Cohort	ICU admission	5,795	1264	1174	58	34.5	53.4		4.5	42.6
**d'Arminio Monforte[170]**	Italy	Prospective cohort	Not valid	539	77	193	66	35.6	46.4	13.7	27.3	17.6
**Dana et al[67]**	France	Cohort	Severe ARDS	222	96	34	64	27.9	48.6	18.9	24.3	26.6
**de Andrade et al[171]**	Brazil	Cross-sectional	Not valid	89405	655	88750	58.9	43.5	4.2			1.5
**Docherty et al[172]**	United Kingdom	Prospective cohort	Not valid	20133	1685	14396	72.9	40.1		16.11	30.9	20.7
**Dreher et al[68]**	Germany	Cohort	ARDS	50	17	4573	65	34	70	50		58
**Eastment et al[69]**	United States	Cohort	ICU admission	25925	12672	4573	60.4±17.0*	10.2	58.6	20.6	18.8	35.4
**Ebinger et al[70]**	United States	Cohort	ICU admission	214	44	76	62	37	54.6	20	21	40
**Fava et al[71]**	Spain	Cohort	ARDS	104	28	76	59.7	42.3	86.5	15.4	29.8	30.8
**Feuth et al[72]**	Finland	Cohort	ICU admission	28	10	17	56	46	43	21		25
**Foulkes et al[73]**	United States	Cohort	Mechanical ventilation	1202	499	592	60.1	43	51	30	15	33
**Fresán et al[74]**	Spain	Prospective cohort	ICU admission	433995	7460	426535		50.1	16.6			
**Friedman et al[75]**	USA	Cohort	IMV	4908	2552	848	60.91	37.2	61.6	10.8	13.4	42.2
**Fusco et al[77]**	United States	Cohort	ICU admission + Mechanical ventilation	173942	46965	126977	63	48.9	64.8	22.2	73.5	40.7
**Feng Gao et al[76]**	China	Cohort	Not mentioned	150	75	75	48	37.8				19.3
**Min Gao et al[93]**	UK	Prospective Cohort Study	ICU admission	13503	4893	3655		44·4	52·7	24·0	26·3	2·0

**Genny Carrillo et al[78]**	Mexico	Retrospective cohort	Mechanical ventilation	69334	16272	53062	55.29	37.38	34.41	5.65	4.21	30.92
**Gerotziafas et al[79]**	France	Cohort	ICU admission	430	67	363	64.3	61	47.7	9		21.6
**Giacomelli et al[173]**	Italy	Cohort	Not valid	233	38	195	61	30.9				
**Giorgi rossi et al[174]**	Italy	Cohort	Not valid	1075	34	1041	63.2	38.8	26	8.4	31	17
**Goncalves et al[175]**	Brazil	Cohort	Not valid	182700	4878	41916		43.4	100	7.5	65.9	55
**Goyal et al[23]**	United States	Cohort	Mechanical ventilation	393	136	244	62.2	39.4	50.1	17.6	13.7	25.2
**Guerson-Gil et al[80]**	United States	Retrospective cohort	Intubation	3530	1472	814	65	44.7	62.9	9.2	8.1	39.8
**Guner et al[81]**	Turkey	Cohort	ARDS, sepsis, and septic shock	222	77	145	50.6	40.5	23.4	5.4	23.6	13.5
**Gupta et al[176]**	United States	Retrospective cohort	Not valid	529	105	133	70	46	79	7	8	56
**Hajifathalian et al[82]**	United States	Cohort	ICU admission	770	277	465	64	39.2	56.1	17.4	21	31
**Halasz et al[177]**	Italy	Cohort	Not valid	242	48	38	64	18.2	45.5	8.7	14.5	15.3
**Halvatsiotis et al[178]**	Greece	Cohort	Not valid	90	31	59	65.5	20	50	12	21.1	18.8
**Hendren et al[33]**	United States	Cohort	Mechanical ventilation	7606	3311	1793	63	45	59.5	9.7	20.6	36.8
**Hojo de Souza et al[179]**	Brazil	Retrospective cohort	Not valid	44128	3633	40495		45.85		6.31	52.02	39.82
**Hsu et al[83]**	United States	Cohort	ICU admission	1088	565	523	63	45	57	24.3	19.7	35.7
**Hur et al[84]**	United States	Cohort	Intubation	486	259	227	59	44.2	54.9	16	22.8	32.9
**Ioannou et al[86]**	United States	Cohort	Mechanical ventilation	3465	1412	1889	61.1	19.6	75	33	47.3	48.8
**Jayanama et al[87]**	Thailand	Cohort	Respiratory rate >30 breaths/min/severe respiratory distress/O2 saturation ≤93 on room air	147	46	56	39.1±13.0*	58.5	9.5			9.5
**John Xie et al[92]**	United States	Retrospective cohort	ICU admission	287	187	100	61.5	56.8	80.1	20.5	14.3	53.6

**Kaeuffer et al**[88]	France	Cohort	Death or admission to ICU	1045	351	236	66.3	41.4	52.4	16.5	11.6	25.3
**Kalligeros et al**[31]	United States	Cohort	ICU admission	103	49	19	60	39.8	64	19.5	24.2	36.8
**Kananen et al**[180]	Sweden	Observational	Not valid	1409	194	709	83	54.3				
**Kang et al**[89]	Korea	Observational	IMV/multi-organ failure/ECMO/death	4141	193	2746		58.3	20.8	2.4	3.2	12.1
**Kates et al**[181]	United States	Cohort	Not valid	482	166	316	57.5	28.8	77.4	10.4	30.1	51
**Nam Hoon Kim et al**[108]	South Korea	Retrospective cohort	ICU admission	4069	191	2206	55.5 ± 14.2*	62.4	32.2	24.9	21.6	14.5
**Tara S. Kim et al**[138]	United States	Cohort	IMV	10861	4090	2507	65	40.5	60.4	8.3	13.3	36.8
**So Young Kim et al**[124]	Korea	Retrospective cohort	O2 via a facial mask or nasal cannula/ventilation/multiorgan failure/ECMO/death	4057	1159	1668		57.5	20.4	2.4	3.3	12.1
**Klang et al**[182]	United States	Cohort	Not valid	3406	1231	2175						
**Kompaniyets et al**[90]	United States	Cohort	ICU admission	148494	75498	28349	55	53.6				
**Emma J. Kooistra et al**[184]	Netherlands	Prospective Cohort	ICU admission	67	18	49		25.4	46.3	8.9	26.9	22.4
**Emma J. Kooistra et al-2**[183]	The Netherlands	Prospective Cohort study	ICU admission	2635	837	592	592	27.9		8.2	1.2	19
**Laccarino et al**[95]	Italy	Cross-sectional	ICU admission	2378	157	28349	68.2	37.4	58.5	8.5	26.4	18.2
**Larvin et al**[185]	United Kingdom	Longitudinal cohort study	Not valid	11999	3654	3234	56.83 ± 8.33*					
**Le Guen et al**[96]	United States	Retrospective cohort	ICU admission	600	301	115	58.9 ± 15.2*	45.5	67	14.7	14.2	42.2
**Lighter et al**[97]	United States	Cohort	ICU admission	3615	1370	2245						
**Ling Hu et al**[94]	China	Cohort	Not mentioned	323	13	229	61	48.6	32.5	10.9	12.7	14.6
**Lodigiani et al**[98]	Italy	Cohort	ICU admission	361	87	130	66	32	47.2			22.7

**Marcello et al[186]**	United States	Cohort	Not valid	6248	2278	1427	61	38	37	11	31	33
**Martín-Del-Campo et al[99]**	Mexico	Retrospective cohort	ICU admission	773	373	121		37.3	47.7	8.2	7.1	37.9
**Mehanna et al[100]**	Egypt	Observational	ICU admission	230	142	30		37.4	28.3			25.2
**Mehta et al[187]**	USA	Retrospective longitudinal cohort study	Not valid	137 119	37318	51 524		67.8		28.8	85.6	36.1
**Mejía-Vilet et al[101]**	Mexico	Cohort	ICU admission	329	132	197	49	36	27			24
**Mendy et al[102]**	United States	Cohort	Death or admission to ICU	216	53	561	60	44.4		16.7	77.3	43.5
**Menezes Soares et al[188]**	Brazil	Cohort	Not valid	1152	111	1039		42.8		9.6	45.6	24
**Mikami et al[189]**	United States	Cohort	Not valid	3708	221	2599	66	43	34.3	8.7		24.3
**Monteiro et al[103]**	United States	Cohort	Mechanical ventilation	112	40	82	61	34	50	17.4	15	64
**Motaib et al[104]**	Morocco	Cohort	ICU admission	107	24	83	53	40	30.8	8.4	15	15
**Mughal et al[105]**	United States	Cohort	Mechanical ventilation	129	18	111	63	37.2	43.3	10.9	17.1	19.4
**Murillo-Zamora et al[190]**	Mexico	Cohort	Not valid	5393	1197	4196		36.4	36.6	7.8		31.1
**Naaraayan et al[191]**	New York, USA	Retrospective cohort study	Not valid	348	121	104	71	43.3	66.7	13.8	32.5	42.2
**Nachega et al[106]**	Democratic Republic of the Congo	Cohort	ICU admission	766	39	725	46	34.4	25.4	3.4	3.9	14
**Nakeshbandi et al[107]**	United States	Cohort	Mechanical ventilation	504	215	139	68	48	83	16	19	53
**Newton et al[109]**	United States	Cohort	Death or admission to ICU	370	102	760	62.2	51.8	66.8	2.5	17.4	42.3

**Okauchi et al[110]**	Japan	Retrospective cohort study	O2 requirement	84	31	51	52.9 ± 19.4*	46.4	32.1			28.6
**Olak et al[192]**	Brazil	Cohort Study	Not valid	728	98	630		40.9			61.7	39.8
**Olivas-Martı´nez et al[193]**	Mexico	Cohort	Not valid	800	357	116	51.9	39	30	2.3	4.6	26
**Oliveira et al[194]**	United States	Retrospective cohort study	Not valid	131	7	40	61	64.9	64.1		9.2	41.2
**Ortiz-Brizuela et al[111]**	Mexico	Cohort	ICU admission	140	50	90	49	29.3	32	2.8	4.3	22.9
**Page-Wilson et al[112]**	United States	Retrospective cohort	ICU admission	1019	420	226	64	41.3	63.5	10.3	13.9	40.1
**Palaiodimos et al[113]**	United States	Cohort	ICU admission	200	46	38	64	51	76	27.5	33.5	39.5
**Parker et al[195]**	South Africa	Cohort	Not valid	113	32	81	48	61	42	17.2	5.4	39
**Parra-Bracamonte et al[196]**	Mexico	Cohort	Not valid	95458	22390	73068	44	38.1	35.1	5.8	4.2	31.3
**Patel et al[197]**	United Kingdom	Cohort	Not valid	1582	520	373	68 ± 9*	47.2	35.5			14.1
**Peña et al[198]**	Mexico	Retrospective Cohort	Not valid	277341	48517	228824		47.8	23.3	2.6	2.2	17.9
**Peng et al[199]**	China	Cohort	Not mentioned	112	33	79	62	59	82		55.3	
**Pepe et al[114]**	multi-country	Cohort	ICU admission	2214	440	1774	49.6	40.2	26.2	12.3	8	9.1
**Petersen, A. et al[115]**	Germany	Cohort	ICU admission	30	19	11	65.6	12	15	3	5	5
**Petrilli et al[116]**	United States	Prospective cohort study	Mechanical ventilation and ICU admission	2741	1081	649	63	38.8	62	16.5	34.8	34.7
**Pettit et al[117]**	United States	Cohort	ICU admission	238	146	43	58.5	52.5	52.9	26.5	21.4	28.6
**Philipose et al[200]**	United Kingdom	Cohort	Not valid	368	125	124	72	40.5	50.2	28.1	33.6	35.4
**Pietri et al[118]**	France	Retrospective cohort	O2 requirement over 6	113	76	37	65.4 ± 15.0*	48.7	51	7	17	19

			L/min/ICU admission/death									
**Plataki et al** [119]	United States	Cohort	Intubation	1337	437	900	65.7	38.6	56.8	9.3	16.6	31.2
**Plourde et al**[120]	Canada	Retrospective cohort	IMV (all critically ill)	94	35	59	60 ±15*	33	46	10		27
**Pongpirul et al**[121]	Thailand	Cohort	severe (respiratory rate >30 breaths/minute, oxygen saturation <93, PaO2/FiO2 ratio <300, and/or lung infiltrates >50 of the lung field within 24–48 hours), and critical (respiratory failure, shock, and/or multiple organ failure)	193	22	99	37	41.5	16.1	1.6	1	8.3
**Pouwels et al**[122]	Netherlands	Retrospective cohort	Sepsis-3, septic shock	121	48	73	68	20.5	30.6	16.5	19	25.6
**Rachel C Frank et al**[123]	United States	Cohort	Mechanical ventilation	305	127	54	60	42	52	18	21	35
**Ramlall et al**[125]	United States	Cohort	Mechanical ventilation	6393	831	5562	57.1	50.3	31.1		26.6	14.2
**Randhawa et al**[126]	United States	Retrospective cohort	Intubation	302	92	210		35.4				37.1
**Rao et al**[127]	China	Cohort	Not mentioned	240	114	127	48	53.8		1.2	17.9	9.6
**Recalde et al**[128]	USA, UK, Spain	Cohort	ICU admission	160013	63866	96147		46.1	72.5	23.8	59.5	48.1
**Reilev et al**[129]	Denmark	Cohort	ICU admission	2254	277	1977	71	38	74	28	45	26
**Rodriguez et al**[201]	Spain	Cohort	Not valid	43	11	32	65.5	27.2	30.2	9.3	14	18.6
**Rodríguez-Molinero et al**[130]	Spain	Cohort	Need for O2 therapy via nonrebreather mask or	418	74	344		57	52		24.1	23.7

			mechanical ventilation									
**Rodriguez-Nava et al[202]**	United States	Retrospective cohort study	Not valid	313	101	212	68	41.9	70.9	21.1	33.5	44.7
**Rottoli et al[131]**	Italy	Cohort	ICU admission	482	104	202	66.2	37.3	51.7	13.1	21.2	15.2
**Serdar Sahin et al[132]**	Turkey	Cross sectional	ICU admission	675	162	238	50.9±17.0*	47	28.1	8.1	10.8	15.6
**Ibrahim Sahin et al[91]**	Turkey	Cohort Study	ICU admission	14625	4337	5068	42	57.4	41.3	22.9	19.2	29.5
**Saito et al[133]**	Japan	Retrospective Cohort	Intubation	580	70	289		33.6	73.3		10.7	39.7
**Salacup G et al[203]**	United States	Cohort	Not valid	242	97	145	66	49.17	74	19.8	14.8	49
**Sardinha et al[204]**	Brazil	Cross-sectional	Not valid	100819	365	100454	42.30 ± 17.60*	51.2		0.6	5.1	4.2
**Schavemaker et al[206]**	The Netherlands	Observational Cohort Study	Not valid	1099	324	244		27	34	7.7	4.4	22.4
**Shah et al[205]**	United States	Cohort	Not valid	522	481	41	63	58.2	79.7	22	22.6	42.3
**Shaikh et al[134]**	Saudi Arabia	Retrospective cohort	ICU admission	565	290	275		24.3	33.8	46.9	17	41.6
**Shekhar et al[135]**	Mexico	Cohort	ICU admission	50	20	19	55.5	54	34	16	14	36
**Silva et al[136]**	Brazil	Cross-sectional	ICU admission	21773	1310	20463		44.7		6.4	52.3	10
**Simonnet et al[137]**	France	Cohort	Mechanical ventilation	124	59	17	60	27	49			23
**Steinberg et al[139]**	United States	Cohort	Mechanical ventilation	210	100	110						
**Suleyman et al[140]**	United States	Cohort	ICU admission	355	210	145	61.4	53.5	72.7	40.3	29.1	43.4
**Suresh et al[141]**	United States	Retrospective cohort	ICU admission	1983	1031	952	63.82 ± 16.55*	50	68	19	29	38
**Tchang et al[142]**	United States	Retrospective cohort	ICU admission or intubation	3533	1256	926	65	41	56	17	15	32
**Tehrani et al[207]**	Sweden	Retrospective cohort	Not valid	255	63	192	66 ± 17*	41	54	13	13	31
**Terada et al[143]**	Japan	Cohort study	Mechanical ventilation/SpO2 ≤94 in room air/tachypnea	3376	178	3194		38.9	18.5	4.1	5.4	16.6

			with respiratory rate ≤24 bpm.									
**Tonetti et al**[144]	Italy	Cohort	ICU admission	700	176	524	69.4	23		14.4	61.8	21.8
**Urra et al**[145]	Spain	Retrospective case control	ICU admission	172	17	155		39.5	50.5	9.8	16.27	22.6
**Vaquero-Roncero et al**[146]	Spain	Cohort	ICU admission	146	46	100						
**Vera-Zertuche et al**[147]	Mexico	Retrospective cohort	ICU admission	15529	2535	10896	46.6 ±15.5*	42.2	21.7	3.5	2.9	18.2
**Wang J et al**[148]	China	Cohort	ICU admission	297	40	140	44.3	44.7	16.16	4.04	2.02	8.41
**Wang Min et al**[150]	China	Retrospective cohort	Respiratory failure	541	60	481	52	45.29	24.77		5.36	8.69
**Wang R et al**[151]	China	Cohort	Not mentioned	96	44	52			52.1			
**Wu et al**[149]	China	Retrospective cohort	ICU admission	1091	285	500	59	53.3	26.4	5.2	7.5	12.6
**Xiang ong et al**[152]	Singapore	Cohort	ICU admission	91	40	51	55	44	33		9.9	19.7
**Yates et al**[153]	UK	Prospective Cohort Study	Not mentioned	412596	98737	137340		55.1				
**Zhang et al**[155]	China	Retrospective cohort	Mechanical ventilation or ICU admission within 30 days	463	42	242		51.6	27.2	3	5.8	11
**Zheng *et al***[156]	China	Cohort	Not mentioned	66	45	21	47	74.2	28.8			24.2
**Zhu et al**[154]	UK	Cohort	Not mentioned	489769	119369	159591	58	54.7	26.8	13.3	13.4	5.1

Table legend: * Mean ± SD, O2: Oxygen, IMV: Invasive mechanical ventilation, ARDS: Acute respiratory distress syndrome, IMV: Intermittent mandatory ventilation, SpO2: Oxygen saturation, ECMO: extracorporeal membrane oxygenation.
