# Supplementary material for: Association of Obesity With COVID-19 Severity and Mortality: An Updated Systemic Review, Meta-Analysis, and Meta-Regression
Source: Front Endocrinol (Lausanne). 2022 Jun 3;13:780872. doi: 10.3389/fendo.2022.780872 (PMC9205425; doi:10.3389/fendo.2022.780872)
Supplement: Supplementary file 3 [file Table_2.pdf]

Table 2: Quality Assessment Tool for Observational Cohort and Cross-Sectional Studies

Reference citation number so for each study correspond to the reference list in the main article

Table 2A: Cohort studies

**Criteria**	**Al Heialy *et al*[45]**	**Al-Sabah *et al*[46]**	**Alkhatib *et al*[48]**	**Anderson *et al*[157]**	**Andrea Rossi *et al*[158]**	**Argenziano *et al*[50]**	**Arjun S *et al*[51]**	**Bellan *et al*[159]**
Was the research question or objective in this paper clearly stated?	Yes	Yes	Yes	Yes	Yes	Yes	Yes	Yes
Was the study population clearly specified and defined?	Yes	Yes	Yes	Yes	Yes	Yes	Yes	Yes
Was the participation rate of eligible persons at least 50%?	Yes	Yes	Yes	Yes	Yes	Yes	No	Yes
Were all the subjects selected or recruited from the same or similar populations (including the same time period)? Were inclusion and exclusion criteria for being in the study prespecified and applied uniformly to all participants?	Yes	Yes	Yes	Yes	Yes	Yes	Yes	Yes
Was a sample size justification, power description, or variance and effect estimates provided?	NR	NR	NR	NR	NR	NR	NR	NR
For the analyses in this paper, was the exposure(s) of interest measured prior to the outcome(s) being measured?	Yes	Yes	Yes	Yes	Yes	Yes	Yes	Yes
Was the timeframe sufficient so that one could reasonably expect to see an association between exposure and outcome if it existed?	No	No	No	No	No	No	No	No
For exposures that can vary in amount or level, did the study examine different levels of the exposure as related to the outcome (e.g., categories of exposure, or exposure measured as continuous variable)?	Yes	Yes	No	Yes	No	No	Yes	NA
Were the exposure measures (independent variables) clearly defined, valid, reliable, and implemented consistently across all study participants?	Yes	Yes	No	Yes	Yes	Yes	Yes	Yes
Was the exposure(s) assessed more than once over time?	NA	NA	No	No	No	No	No	No
Were the outcome measures (dependent variables) clearly defined, valid, reliable, and implemented consistently across all study participants?	Yes	Yes	No	Yes	Yes	NA	NA	NA
Were the outcome assessors blinded to the exposure status of participants?	No	No	No	No	No	No	No	No
Was loss to follow-up after baseline 20% or less?	Yes	Yes	Yes	Yes	Yes	Yes	Yes	Yes
Were key potential confounding variables measured and adjusted statistically for their impact on the relationship between exposure(s) and outcome(s)?	Yes	Yes	Yes	Yes	Yes	Yes	No	Yes
**Quality Rating (Good, Fair, or Poor)**	Good	Good	Low	Good	Good	Good	Low	Good
**Risk of Bias**	Low	Low	Moderate	Low	Low	Low	Moderate	Low

CD, cannot determine; NA, not applicable; NR, not reported

Table 2A: Cohort studies (continued)

**Criteria**	**Biscarini *et al*[54]**	**Borobia *et al*[161]**	**Burrell *et al*[55]**	**Busetto *et al*[56]**	**Cai *et al*[57]**	**Cariou *et al*[162]**	**Cravedi *et al*[168]**
Was the research question or objective in this paper clearly stated?	Yes	Yes	Yes	Yes	Yes	Yes	Yes
Was the study population clearly specified and defined?	Yes	Yes	Yes	Yes	Yes	Yes	Yes
Was the participation rate of eligible persons at least 50%?	Yes	Yes	Yes	Yes	Yes	Yes	Yes
Were all the subjects selected or recruited from the same or similar populations (including the same time period)? Were inclusion and exclusion criteria for being in the study prespecified and applied uniformly to all participants?	Yes	Yes	Yes	Yes	Yes	Yes	Yes
Was a sample size justification, power description, or variance and effect estimates provided?	NR	NR	NR	NR	NR	NR	NR
For the analyses in this paper, was the exposure(s) of interest measured prior to the outcome(s) being measured?	Yes	Yes	Yes	No	Yes	Yes	Yes
Was the timeframe sufficient so that one could reasonably expect to see an association between exposure and outcome if it existed?	No	No	No	No	No	NA	No
For exposures that can vary in amount or level, did the study examine different levels of the exposure as related to the outcome (e.g., categories of exposure, or exposure measured as continuous variable)?	Yes	Yes	No	Yes	NA	CD	Yes
Were the exposure measures (independent variables) clearly defined, valid, reliable, and implemented consistently across all study participants?	Yes	Yes	Yes	Yes	Yes	Yes	Yes
Was the exposure(s) assessed more than once over time?	Yes	Yes	Yes	Yes	Yes	Yes	No
Were the outcome measures (dependent variables) clearly defined, valid, reliable, and implemented consistently across all study participants?	NA	Na	NA	Yes	NA	Yes	Yes
Were the outcome assessors blinded to the exposure status of participants?	No	No	No	No	No	No	No
Was loss to follow-up after baseline 20% or less?	Yes	Yes	Yes	NA	Yes	Yes	Yes
Were key potential confounding variables measured and adjusted statistically for their impact on the relationship between exposure(s) and outcome(s)?	Yes	Yes	No	Yes	Yes	Yes	Yes
**Quality Rating (Good, Fair, or Poor)**	High	High	Good	Low	Good	Good	Good
**Risk of Bias**	Very low	Very low	Low	Moderate	Low	Low	Low

CD, cannot determine; NA, not applicable; NR, not reported

Table 2A: Cohort studies (continued)

Criteria	Carrillo-Vega *et al*[163]	Castelnuova *et al*[164]	Caussy *et al*[60]	Cedano *et al*[165]	Chand *et al*[166]	Claudia Gregoriano et al[63]	Czernichow *et al*[66]
Was the research question or objective in this paper clearly stated?	Yes	Yes	Yes	Yes	Yes	Yes	Yes
Was the study population clearly specified and defined?	Yes	Yes	Yes	Yes	Yes	Yes	Yes
Was the participation rate of eligible persons at least 50%?	Yes	Yes	yes	Yes	Yes	Yes	Yes
Were all the subjects selected or recruited from the same or similar populations (including the same time period)? Were inclusion and exclusion criteria for being in the study prespecified and applied uniformly to all participants?	Yes	Yes	Yes	Yes	Yes	Yes	Yes
Was a sample size justification, power description, or variance and effect estimates provided?	NR	NR	NR	NR	NR	NR	NR
For the analyses in this paper, was the exposure(s) of interest measured prior to the outcome(s) being measured?	Yes	Yes	No	Yes	Yes	Yes	Yes
Was the timeframe sufficient so that one could reasonably expect to see an association between exposure and outcome if it existed?	No	NO	NA	No	No	No	No
For exposures that can vary in amount or level, did the study examine different levels of the exposure as related to the outcome (e.g., categories of exposure, or exposure measured as continuous variable)?	No	Yes	CD	Yes	Yes	Yes	CD
Were the exposure measures (independent variables) clearly defined, valid, reliable, and implemented consistently across all study participants?	Yes	Yes	Yes	Yes	Yes	Yes	Yes
Was the exposure(s) assessed more than once over time?	No	Yes	Yes	Yes	Yes	Yes	Yes
Were the outcome measures (dependent variables) clearly defined, valid, reliable, and implemented consistently across all study participants?	Yes	Yes	CD	Yes	NA	Na	NA
Were the outcome assessors blinded to the exposure status of participants?	No	No	No	No	No	No	No
Was loss to follow-up after baseline 20% or less?	Yes	NA	Yes	NA	Yes	Yes	Yes
Were key potential confounding variables measured and adjusted statistically for their impact on the relationship between exposure(s) and outcome(s)?	Yes	Yes	Yes	Yes	Yes	Yes	Yes
**Quality Rating (Good, Fair, or Poor)**	Good	Good	Low	Good	Good	High	Good
**Risk of Bias**	Low	Low	Moderate	Low	Low	Very low	Low

CD, cannot determine; NA, not applicable; NR, not reported

Table 2A: Cohort studies (continued)

Criteria	de Andrade et al[171]	Docherty et al[172]	Dreher et al[68]	Ebinger et al[70]	Fava et al[71]	Feuth et al[72]	Fusco et al[77]	Feng Gao *et al*[76]
Was the research question or objective in this paper clearly stated?	Yes	Yes	Yes	Yes	Yes	Yes	Yes	Yes
Was the study population clearly specified and defined?	Yes	Yes	Yes	Yes	Yes	Yes	Yes	Yes
Was the participation rate of eligible persons at least 50%?	Yes	Yes	Yes	Yes	Yes	Yes	Yes	Yes
Were all the subjects selected or recruited from the same or similar populations (including the same time period)? Were inclusion and exclusion criteria for being in the study prespecified and applied uniformly to all participants?	Yes	Yes	Yes	Yes	Yes	Yes	Yes	Yes
Was a sample size justification, power description, or variance and effect estimates provided?	NR	NR	NR	NR	NR	NR	NR	NR
For the analyses in this paper, was the exposure(s) of interest measured prior to the outcome(s) being measured?	No	Yes	Yes	Yes	Yes	Yes	Yes	Yes
Was the timeframe sufficient so that one could reasonably expect to see an association between exposure and outcome if it existed?	No	No	No	No	No	No	No	NO
For exposures that can vary in amount or level, did the study examine different levels of the exposure as related to the outcome (e.g., categories of exposure, or exposure measured as continuous variable)?	No	Yes	Yes	No	No	Yes	Yes	Yes
Were the exposure measures (independent variables) clearly defined, valid, reliable, and implemented consistently across all study participants?	Yes	Yes	No	Yes	No	No	No	No
Was the exposure(s) assessed more than once over time?	Yes	Yes	Yes	Yes	Yes	Yes	No	No
Were the outcome measures (dependent variables) clearly defined, valid, reliable, and implemented consistently across all study participants?	Yes	Yes	Yes	Yes	Yes	Yes	Yes	Yes
Were the outcome assessors blinded to the exposure status of participants?	No	No	No	No	No	No	No	No
Was loss to follow-up after baseline 20% or less?	Yes	Yes	Yes	Yes	Yes	Yes	Yes	Yes
Were key potential confounding variables measured and adjusted statistically for their impact on the relationship between exposure(s) and outcome(s)?	Yes	Yes	Yes	Yes	Yes	Yes	Yes	Yes
**Quality Rating (Good, Fair, or Poor)**	Good	High	Good	Good	Good	Good	Good	Good
**Risk of Bias**	Low	Very low	Low	Low	Low	Low	Low	Low

CD, cannot determine; NA, not applicable; NR, not reported

Table 2A: Cohort studies (continued)

Criteria	Genny Carrillo *et al*[78]	Gerotziafas *et al*[79]	Giacomelli *et al*[173]	Giorgi rossi *et al*[174]	Goyal et al[23]	Guner *et al*[81]	Hajifathalian et al[82]	Halasz et al[177]
Was the research question or objective in this paper clearly stated?	Yes	Yes	Yes	Yes	Yes	Yes	Yes	YEs
Was the study population clearly specified and defined?	Yes	Yes	yes	Yes	Yes	Yes	Yes	Yes
Was the participation rate of eligible persons at least 50%?	Yes	Yes	yes	Yes	Yes	Yes	Yes	Yes
Were all the subjects selected or recruited from the same or similar populations (including the same time period)? Were inclusion and exclusion criteria for being in the study prespecified and applied uniformly to all participants?	Yes	Yes	Yes	Yes	Yes	Yes	Yes	Yes
Was a sample size justification, power description, or variance and effect estimates provided?	NR	NR	NR	NR	NR	NR	NR	NR
For the analyses in this paper, was the exposure(s) of interest measured prior to the outcome(s) being measured?	Yes	Yes	Yes	Yes	NO	Yes	Yes	Yes
Was the timeframe sufficient so that one could reasonably expect to see an association between exposure and outcome if it existed?	No	Yes	Yes	Yes	No	No	No	No
For exposures that can vary in amount or level, did the study examine different levels of the exposure as related to the outcome (e.g., categories of exposure, or exposure measured as continuous variable)?	Yes	No	No	No	No	Yes	Yes	Yes
Were the exposure measures (independent variables) clearly defined, valid, reliable, and implemented consistently across all study participants?	NO	Yes	No	No	Yes	No	Yes	Yes
Was the exposure(s) assessed more than once over time?	No	Yes	Yes	No	No	No	Yes	NO
Were the outcome measures (dependent variables) clearly defined, valid, reliable, and implemented consistently across all study participants?	Yes	Yes	Yes	Yes	Yes	Yes	Yes	Yes
Were the outcome assessors blinded to the exposure status of participants?	No	No	No	No	No	No	No	No
Was loss to follow-up after baseline 20% or less?	Yes	Yes	Yes	Yes	Yes	Yes	Yes	Yes
Were key potential confounding variables measured and adjusted statistically for their impact on the relationship between exposure(s) and outcome(s)?	No	No	No	Yes	No	Yes	Yes	Yes
**Quality Rating (Good, Fair, or Poor)**	Fair	Fair	Good	Good	Low	Good	High	Good
**Risk of Bias**	Unclear	Unclear	Low	Low	Moderate	Low	Very low	Low

CD, cannot determine; NA, not applicable; NR, not reported

Table 2A: Cohort studies (continued)

**Criteria**	**Halvatsiotis *et al*[178]**	**Hojo de Souza et al[179]**	**Hsu et al[83]**	**Hur et al[84]**	**Laccarino et al[95]**	**Ioannou et al[86]**	**John Xie et al[92]**	**Kaeuffer et al[88]**
Was the research question or objective in this paper clearly stated?	Yes	Yes	Yes	Yes	Yes	Yes	Yes	Yes
Was the study population clearly specified and defined?	Yes	Yes	Yes	Yes	Yes	Yes	Yes	Yes
Was the participation rate of eligible persons at least 50%?	Yes	Yes	Yes	Yes	Yes	Yes	Yes	Yes
Were all the subjects selected or recruited from the same or similar populations (including the same time period)? Were inclusion and exclusion criteria for being in the study prespecified and applied uniformly to all participants?	Yes	Yes	Yes	Yes	Yes	Yes	Yes	Yes
Was a sample size justification, power description, or variance and effect estimates provided?	NR	NR	NR	NR	NR	NR	NR	NR
For the analyses in this paper, was the exposure(s) of interest measured prior to the outcome(s) being measured?	Yes	Yes	Yes	Yes	No	Yes	Yes	Yes
Was the timeframe sufficient so that one could reasonably expect to see an association between exposure and outcome if it existed?	No	No	No	No	No	No	No	No
For exposures that can vary in amount or level, did the study examine different levels of the exposure as related to the outcome (e.g., categories of exposure, or exposure measured as continuous variable)?	Yes	Yes	Yes	Yes	Yes	Yes	Yes	Yes
Were the exposure measures (independent variables) clearly defined, valid, reliable, and implemented consistently across all study participants?	Yes	Yes	No	Yes	Yes	Yes	Yes'	Yes
Was the exposure(s) assessed more than once over time?	No	No	Yes	No	No	Yes	Yes	Yes
Were the outcome measures (dependent variables) clearly defined, valid, reliable, and implemented consistently across all study participants?	Yes	Yes	Yes	Yes	Yes	Yes	Yes	Yes
Were the outcome assessors blinded to the exposure status of participants?	No	No	No	No	No	No	No	No
Was loss to follow-up after baseline 20% or less?	Yes	Yes	Yes	Yes	Yes	Yes	Yes	Yes
Were key potential confounding variables measured and adjusted statistically for their impact on the relationship between exposure(s) and outcome(s)?	Yes	Yes	No	Yes	NA	Yes	CD	Yes
**Quality Rating (Good, Fair, or Poor)**	Good	Good	Good	Good	Good	High	High	High
**Risk of Bias**	Low	Low	Low	Low	Low	Very low	Very low	Very low

CD, cannot determine; NA, not applicable; NR, not reported

Table 2A: Cohort studies (continued)

Criteria	Kalligeros et al[31]	Kates et al[181]	Klang et al[182]	Lighter et al[97]	Ling Hu et al[94]	Lodigiani et al[98]	Marcello et al[186]	Mejía-Vilet et al[101]
Was the research question or objective in this paper clearly stated?	Yes	Yes	Yes	Yes	Yes	Yes	Yes	Yes
Was the study population clearly specified and defined?	Yes	Yes	Yes	Yes	Yes	Yes	Yes	Yes
Was the participation rate of eligible persons at least 50%?	Yes	Yes	Yes	Yes	Yes	Yes	Yes	Yes
Were all the subjects selected or recruited from the same or similar populations (including the same time period)? Were inclusion and exclusion criteria for being in the study prespecified and applied uniformly to all participants?	Yes	Yes	Yes	Yes	Yes	Yes	Yes	Yes
Was a sample size justification, power description, or variance and effect estimates provided?	NR	NR	NR	NR	NR	NR	NR	NR
For the analyses in this paper, was the exposure(s) of interest measured prior to the outcome(s) being measured?	Yes	Yes	Yes	Yes	Yes	Yes	Yes	Yes
Was the timeframe sufficient so that one could reasonably expect to see an association between exposure and outcome if it existed?	No	No	No	No	CD	CD	No	No
For exposures that can vary in amount or level, did the study examine different levels of the exposure as related to the outcome (e.g., categories of exposure, or exposure measured as continuous variable)?	Yes	Yes	Yes	Yes	Yes	Yes	Yes	Yes
Were the exposure measures (independent variables) clearly defined, valid, reliable, and implemented consistently across all study participants?	Yes	Yes	No	No	Yes	Yes	Yes	Yes
Was the exposure(s) assessed more than once over time?	Yes	No	Yes	No	No	Yes	No	Yes
Were the outcome measures (dependent variables) clearly defined, valid, reliable, and implemented consistently across all study participants?	Yes	Yes	Yes	Yes	Yes	Yes	Yes	Yes
Were the outcome assessors blinded to the exposure status of participants?	No	No	No	No	No	No	No	No
Was loss to follow-up after baseline 20% or less?	Yes	Yes	Yes	Yes	Yes	Yes	Yes	Yes
Were key potential confounding variables measured and adjusted statistically for their impact on the relationship between exposure(s) and outcome(s)?	Yes	Yes	Yes	No	Yes	Yes	Yes	Yes
**Quality Rating**	High	Good	Good	Fair	High	Good	Good	High
**Risk of Bias**	Very low	Low	Low	Unclear	Very low	Low	Low	Very low

CD, cannot determine; NA, not applicable; NR, not reported

Table 2A: Cohort studies (continued)

Criteria	Mendy *et al*[102]	Menezes Soares *et al*[188]	Mikami *et al*[189]	Monteiro et al[103]	Motaib et al[104]	Mughal *et al*[105]	Murillo-Zamora *et al*[190]	Nachega et al[106]
Was the research question or objective in this paper clearly stated?	Yes	Yes	Yes	Yes	Yes	Yes	Yes	Yes
Was the study population clearly specified and defined?	Yes	Yes	Yes	Yes	Yes	Yes	Yes	Yes
Was the participation rate of eligible persons at least 50%?	Yes	Yes	Yes	Yes	Yes	Yes	Yes	Yes
Were all the subjects selected or recruited from the same or similar populations (including the same time period)? Were inclusion and exclusion criteria for being in the study prespecified and applied uniformly to all participants?	Yes	Yes	Yes	Yes	Yes	Yes	Yes	Yes
Was a sample size justification, power description, or variance and effect estimates provided?	NR	NR	NR	NR	NR	NR	NR	NR
For the analyses in this paper, was the exposure(s) of interest measured prior to the outcome(s) being measured?	Yes	Yes	Yes	Yes	Yes	Yes	Yes	Yes
Was the timeframe sufficient so that one could reasonably expect to see an association between exposure and outcome if it existed?	Yes	No	No	No	No	Yes	No	No
For exposures that can vary in amount or level, did the study examine different levels of the exposure as related to the outcome (e.g., categories of exposure, or exposure measured as continuous variable)?	Yes	Yes	Yes	Yes	Yes	Yes	Yes	Yes
Were the exposure measures (independent variables) clearly defined, valid, reliable, and implemented consistently across all study participants?	Yes	Yes	Yes	Yes	Yes	Yes	Yes	Yes
Was the exposure(s) assessed more than once over time?	No	Yes	Yes	Yes	No	Yes	Yes	No
Were the outcome measures (dependent variables) clearly defined, valid, reliable, and implemented consistently across all study participants?	Yes	Yes	Yes	Yes	Yes	Yes	Yes	Yes
Were the outcome assessors blinded to the exposure status of participants?	No	No	No	No	No	No	No	No
Was loss to follow-up after baseline 20% or less?	Yes	Yes	Yes	Yes	Yes	Yes	Yes	Yes
Were key potential confounding variables measured and adjusted statistically for their impact on the relationship between exposure(s) and outcome(s)?	Yes	Yes	Yes	Yes	Yes	Yes	Yes	Yes
**Quality Rating**	High	High	Good	High	Good	High	High	Good
**Risk of Bias**	Very low	Very low	Low	Very low	Low	Very low	Very low	Low

CD, cannot determine; NA, not applicable; NR, not reported

Table 2A: Cohort studies (continued)

Criteria	Nakeshbandi *et al*[107]	Newton *et al*[109]	Ortiz-Brizuela *et al*[111]	Palaiodimos *et al*[113]	Parker *et al*[195]	Parra-Bracamonte et al[196]	Peng et al[199]
Was the research question or objective in this paper clearly stated?	Yes	Yes	Yes	Yes	Yes	Yes	Yes
Was the study population clearly specified and defined?	Yes	Yes	Yes	Yes	Yes	Yes	Yes
Was the participation rate of eligible persons at least 50%?	Yes	Yes	Yes	Yes	Yes	Yes	Yes
Were all the subjects selected or recruited from the same or similar populations (including the same time period)? Were inclusion and exclusion criteria for being in the study prespecified and applied uniformly to all participants?	Yes	Yes	Yes	Yes	Yes	Yes	Yes
Was a sample size justification, power description, or variance and effect estimates provided?	NR	NR	NR	NR	NR	NR	NR
For the analyses in this paper, was the exposure(s) of interest measured prior to the outcome(s) being measured?	Yes	No	Yes	Yes	Yes	Yes	Yes
Was the timeframe sufficient so that one could reasonably expect to see an association between exposure and outcome if it existed?	No	No	No	No	No	No	No
For exposures that can vary in amount or level, did the study examine different levels of the exposure as related to the outcome (e.g., categories of exposure, or exposure measured as continuous variable)?	Yes	Yes	Yes	Yes	Yes	Yes	Yes
Were the exposure measures (independent variables) clearly defined, valid, reliable, and implemented consistently across all study participants?	Yes	Yes	No	Yes	Yes	Yes	Yes
Was the exposure(s) assessed more than once over time?	No	Yes	Yes	CD	CD	No	No
Were the outcome measures (dependent variables) clearly defined, valid, reliable, and implemented consistently across all study participants?	Yes	Yes	Yes	Yes	Yes	Yes	Yes
Were the outcome assessors blinded to the exposure status of participants?	No	No	No	No	No	No	No
Was loss to follow-up after baseline 20% or less?	Yes	Yes	Yes	Yes	Yes	Yes	Yes
Were key potential confounding variables measured and adjusted statistically for their impact on the relationship between exposure(s) and outcome(s)?	Yes	Yes	CD	Yes	CD	Yes	Yes
**Quality Rating**	Good	High	Good	High	Good	Good	Good
**Risk of Bias**	Low	Very low	Low	Very low	Low	Low	Low

CD, cannot determine; NA, not applicable; NR, not reported

Table 2A: Cohort studies (continued)

Criteria	Pepe et al[114]	Petersen, A. et al[115]	Petrilli et al[116]	Pettit *et al*[117]	Philipose *et al*[200]	Pongpirul et al[121]	Rachel C Frank et al[123]	Ramlall *et al*[125]
Was the research question or objective in this paper clearly stated?	Yes	Yes	Yes	Yes	Yes	Yes	Yes	Yes
Was the study population clearly specified and defined?	Yes	Yes	Yes	Yes	Yes	Yes	Yes	Yes
Was the participation rate of eligible persons at least 50%?	Yes	Yes	Yes	Yes	Yes	Yes	Yes	Yes
Were all the subjects selected or recruited from the same or similar populations (including the same time period)? Were inclusion and exclusion criteria for being in the study prespecified and applied uniformly to all participants?	Yes	Yes	Yes	Yes	Yes	Yes	Yes	Yes
Was a sample size justification, power description, or variance and effect estimates provided?	NR	NR	NR	NR	NR	NR	NR	NR
For the analyses in this paper, was the exposure(s) of interest measured prior to the outcome(s) being measured?	Yes	Yes	Yes	Yes	Yes	Yes	Yes	Yes
Was the timeframe sufficient so that one could reasonably expect to see an association between exposure and outcome if it existed?	No	No	No	No	No	Yes	CD	No
For exposures that can vary in amount or level, did the study examine different levels of the exposure as related to the outcome (e.g., categories of exposure, or exposure measured as continuous variable)?	Yes	Yes	Yes	Yes	Yes	Yes	Yes	Yes
Were the exposure measures (independent variables) clearly defined, valid, reliable, and implemented consistently across all study participants?	Yes	Yes	Yes	Yes	Yes	Yes	Yes	Yes
Was the exposure(s) assessed more than once over time?	No	Yes	No	No	No	Yes	Yes	NO
Were the outcome measures (dependent variables) clearly defined, valid, reliable, and implemented consistently across all study participants?	Yes	Yes	Yes	Yes	Yes	Yes	Yes	Yes
Were the outcome assessors blinded to the exposure status of participants?	No	No	No	No	No	No	No	No
Was loss to follow-up after baseline 20% or less?	Yes	Yes	Yes	Yes	Yes	Yes	Yes	Yes
Were key potential confounding variables measured and adjusted statistically for their impact on the relationship between exposure(s) and outcome(s)?	Yes	Yes	Yes	CD	Yes	Yes	Yes	Yes
**Quality Rating**	Good	High	Good	Good	Good	High	High	Good
**Risk of Bias**	Low	Very low	Low	Low	Low	Very low	Very low	Low

CD, cannot determine; NA, not applicable; NR, not reported

Table 2A: Cohort studies (continued)

**Criteria**	**Rao et al**[127]	**Reilev et al**[129]	**Rodriguez et al**[201]	**Rodríguez-Molinero et al**[130]	**Salacup G *et al***[203]	**Rottoli *et al***[131]	**Shah et al**[205]	**Shekhar et al**[135]	**Simonnet et al**[137]
Was the research question or objective in this paper clearly stated?	Yes	Yes	Yes	Yes	Yes	Yes	Yes	Yes	Yes
Was the study population clearly specified and defined?	Yes	Yes	Yes	Yes	Yes	Yes	Yes	Yes	Yes
Was the participation rate of eligible persons at least 50%?	Yes	Yes	Yes	Yes	Yes	Yes	Yes	Yes	Yes
Were all the subjects selected or recruited from the same or similar populations (including the same time period)? Were inclusion and exclusion criteria for being in the study prespecified and applied uniformly to all participants?	Yes	Yes	Yes	Yes	Yes	Yes	Yes	Yes	Yes
Was a sample size justification, power description, or variance and effect estimates provided?	NR	NR	NR	NR	NR	NR	NR	NR	NR
For the analyses in this paper, was the exposure(s) of interest measured prior to the outcome(s) being measured?	Yes	Yes	Yes	Yes	No	Yes	Yes	Yes	Yes
Was the timeframe sufficient so that one could reasonably expect to see an association between exposure and outcome if it existed?	CD	No	CD	No	Yes	No	No	No	No
For exposures that can vary in amount or level, did the study examine different levels of the exposure as related to the outcome (e.g., categories of exposure, or exposure measured as continuous variable)?	Yes	Yes	Yes	Yes	Yes	Yes	Yes	Yes	Yes
Were the exposure measures (independent variables) clearly defined, valid, reliable, and implemented consistently across all study participants?	Yes	Yes	Yes	Yes	Yes	No	Yes	Yes	Yes
Was the exposure(s) assessed more than once over time?	No	Yes	No	No	No	No	No	No	No
Were the outcome measures (dependent variables) clearly defined, valid, reliable, and implemented consistently across all study participants?	Yes	No	Yes	Yes	Yes	Yes	Yes	Yes	Yes
Were the outcome assessors blinded to the exposure status of participants?	No	No	No	No	No	No	No	No	No
Was loss to follow-up after baseline 20% or less?	Yes	Yes	Yes	Yes	Yes	Yes	Yes	Yes	Yes
Were key potential confounding variables measured and adjusted statistically for their impact on the relationship between exposure(s) and outcome(s)?	Yes	Yes	Yes	Yes	Yes	Yes	Yes	Yes	Yes
**Quality Rating**	High	Good	High	Good	Good	Good	High	Good	High
**Risk of Bias**	Very low	Low	Very low	Low	Low	Low	Very low	Low	Very low

CD, cannot determine; NA, not applicable; NR, not reported

Table 2A: Cohort studies (continued)

**Criteria**	**Steinberg *et al*[139]**	**Suleyman *et al*[140]**	**Tonetti et al[144]**	**Vaquero - Roncero et al[146]**	**Wang J et al[148]**	**Wang Min et al[150]**	**Wang R *et al*[151]**	**Xiang ong et al[152]**	**Zheng *et al*[156]**
Was the research question or objective in this paper clearly stated?	Yes	Yes	Yes	Yes	Yes	Yes	Yes	Yes	Yes
Was the study population clearly specified and defined?	Yes	Yes	Yes	Yes	Yes	Yes	Yes	Yes	Yes
Was the participation rate of eligible persons at least 50%?	Yes	Yes	Yes	Yes	Yes	Yes	Yes	Yes	Yes
Were all the subjects selected or recruited from the same or similar populations (including the same time period)? Were inclusion and exclusion criteria for being in the study prespecified and applied uniformly to all participants?	Yes	Yes	Yes	Yes	Yes	Yes	Yes	Yes	Yes
Was a sample size justification, power description, or variance and effect estimates provided?	NR	NR	NR	NR	NR	NR	NR	NR	NR
For the analyses in this paper, was the exposure(s) of interest measured prior to the outcome(s) being measured?	Yes	No	Yes	Yes	Yes	Yes	Yes	Yes	Yes
Was the timeframe sufficient so that one could reasonably expect to see an association between exposure and outcome if it existed?	NO	No	No	No	No	NO	No	No	No
For exposures that can vary in amount or level, did the study examine different levels of the exposure as related to the outcome (e.g., categories of exposure, or exposure measured as continuous variable)?	Yes	Yes	Yes	Yes	Yes	Yes	yes	Yes	Yes
Were the exposure measures (independent variables) clearly defined, valid, reliable, and implemented consistently across all study participants?	Yes	Yes	Yes	Yes	Yes	Yes	Yes	Yes	Yes
Was the exposure(s) assessed more than once over time?	No	No	No	No	NO	No	No	No	No
Were the outcome measures (dependent variables) clearly defined, valid, reliable, and implemented consistently across all study participants?	Yes	Yes	Yes	Yes	No	Yes	Yes	Yes	Yes
Were the outcome assessors blinded to the exposure status of participants?	No	No	No	No	No	No	No	No	No
Was loss to follow-up after baseline 20% or less?	Yes	Yes	Yes	Yes	Yes	Yes	Yes	Yes	Yes
Were key potential confounding variables measured and adjusted statistically for their impact on the relationship between exposure(s) and outcome(s)?	No	Yes	Yes	Yes	Yes	CD	CD	CD	Yes
**Quality Rating**	Good	Good	Good	High	Good	Good	Good	Good	Good
**Risk of Bias**	Low	Low	Low	Very low	Low	Low	Low	Low	Low

CD, cannot determine; NA, not applicable; NR, not reported

Table 2A: Cohort studies (continued)

Criteria	Abumayyaleh et al[43]	Agca et al[44]	Al-Salameh et al[47]	Ando et al[49]	Bailly et al[52]	Bellini et al[160]	Cao et al[58]	Castilla et al[59]
Was the research question or objective in this paper clearly stated?	Yes	Yes	Yes	Yes	Yes	Yes	Yes	Yes
Was the study population clearly specified and defined?	Yes	Yes	Yes	Yes	Yes	Yes	Yes	Yes
Was the participation rate of eligible persons at least 50%?	No	Yes	Yes	Yes	Yes	Yes	Yes	No
Were all the subjects selected or recruited from the same or similar populations (including the same time period)? Were inclusion and exclusion criteria for being in the study prespecified and applied uniformly to all participants?	Yes	Yes	Yes	Yes	Yes	Yes	Yes	Yes
Was a sample size justification, power description, or variance and effect estimates provided?	NR	NR	NR	NR	NR	NR	NR	NR
For the analyses in this paper, was the exposure(s) of interest measured prior to the outcome(s) being measured?	Yes	No	Yes	Yes	Yes	Yes	Yes	Yes
Was the timeframe sufficient so that one could reasonably expect to see an association between exposure and outcome if it existed?	Yes	No	Yes	Yes	Yes	Yes	Yes	Yes
For exposures that can vary in amount or level, did the study examine different levels of the exposure as related to the outcome (e.g., categories of exposure, or exposure measured as continuous variable)?	Yes	Yes	Yes	No	No	No	Yes	No
Were the exposure measures (independent variables) clearly defined, valid, reliable, and implemented consistently across all study participants?	Yes	Yes	Yes	No	No	CD	Yes	No
Was the exposure(s) assessed more than once over time?	No	No	No	No	No	No	No	No
Were the outcome measures (dependent variables) clearly defined, valid, reliable, and implemented consistently across all study participants?	Yes	Yes	Yes	Yes	Yes	Yes	Yes	Yes
Were the outcome assessors blinded to the exposure status of participants?	No	No	No	No	No	No	No	No
Was loss to follow-up after baseline 20% or less?	Yes	Yes	Yes	Yes	Yes	Yes	Yes	Yes
Were key potential confounding variables measured and adjusted statistically for their impact on the relationship between exposure(s) and outcome(s)?	Yes	Yes	Yes	Yes	Yes	Yes	Yes	Yes
**Quality Rating**	Good	Fair	Good	Fair	Fair	Fair	Good	Low
**Risk of Bias**	Low	Unclear	Low	Unclear	Unclear	Unclear	Low	Moderate

CD, cannot determine; NA, not applicable; NR, not reported

Table 2A: Cohort studies (continued)

Criteria	Chetboun et al[61]	Cho et al[62]	Ciceri et al[167]	Coss-Rovirosa et al[64]	Cottini et al[65]	Cueto-Manzano et al[169]	d'Arminio Monforte[170]	Dana et al[67]
Was the research question or objective in this paper clearly stated?	Yes	Yes	Yes	Yes	Yes	Yes	Yes	Yes
Was the study population clearly specified and defined?	Yes	Yes	Yes	Yes	Yes	Yes	Yes	Yes
Was the participation rate of eligible persons at least 50%?	CD	No	Yes	NR	Yes	Yes	Yes	Yes
Were all the subjects selected or recruited from the same or similar populations (including the same time period)? Were inclusion and exclusion criteria for being in the study prespecified and applied uniformly to all participants?	Yes	Yes	Yes	Yes	Yes	Yes	Yes	Yes
Was a sample size justification, power description, or variance and effect estimates provided?	NR	NR	NR	NR	NR	NR	NR	NR
For the analyses in this paper, was the exposure(s) of interest measured prior to the outcome(s) being measured?	Yes	Yes	Yes	Yes	Yes	Yes	Yes	Yes
Was the timeframe sufficient so that one could reasonably expect to see an association between exposure and outcome if it existed?	Yes	Yes	Yes	Yes	Yes	Yes	Yes	Yes
For exposures that can vary in amount or level, did the study examine different levels of the exposure as related to the outcome (e.g., categories of exposure, or exposure measured as continuous variable)?	Yes	Yes	Yes	Yes	Yes	Yes	No	Yes
Were the exposure measures (independent variables) clearly defined, valid, reliable, and implemented consistently across all study participants?	Yes	Yes	Yes	Yes	Yes	Yes	No	Yes
Was the exposure(s) assessed more than once over time?	No	No	No	No	No	No	No	No
Were the outcome measures (dependent variables) clearly defined, valid, reliable, and implemented consistently across all study participants?	Yes	Yes	Yes	Yes	Yes	Yes	Yes	Yes
Were the outcome assessors blinded to the exposure status of participants?	No	No	No	No	No	No	No	No
Was loss to follow-up after baseline 20% or less?	Yes	Yes	Yes	Yes	Yes	Yes	Yes	Yes
Were key potential confounding variables measured and adjusted statistically for their impact on the relationship between exposure(s) and outcome(s)?	Yes	Yes	Yes	Yes	Yes	Yes	Yes	Yes
**Quality Rating**	Good	Good	Good	Good	Good	Good	Fair	Good
**Risk of Bias**	Low	Low	Low	Low	Low	Low	Unclear	Low

CD, cannot determine; NA, not applicable; NR, not reported

Table 2A: Cohort studies (continued)

Criteria	Eastment et al[69]	Foulkes et al[73]	Fresán et al[74]	Friedman et al[75]	Goncalves et al[175]	Guerson-Gil et al[80]	Gupta et al[176]	Hendren et al[33]
Was the research question or objective in this paper clearly stated?	Yes	Yes	Yes	Yes	Yes	Yes	Yes	Yes
Was the study population clearly specified and defined?	Yes	Yes	Yes	Yes	Yes	Yes	Yes	Yes
Was the participation rate of eligible persons at least 50%?	Yes	NR	Yes	Yes	NR	NR	Yes	Yes
Were all the subjects selected or recruited from the same or similar populations (including the same time period)? Were inclusion and exclusion criteria for being in the study prespecified and applied uniformly to all participants?	Yes	Yes	Yes	Yes	Yes	Yes	Yes	Yes
Was a sample size justification, power description, or variance and effect estimates provided?	NR	NR	NR	NR	NR	NR	NR	NR
For the analyses in this paper, was the exposure(s) of interest measured prior to the outcome(s) being measured?	Yes	Yes	Yes	Yes	Yes	Yes	Yes	Yes
Was the timeframe sufficient so that one could reasonably expect to see an association between exposure and outcome if it existed?	Yes	Yes	Yes	Yes	Yes	Yes	Yes	Yes
For exposures that can vary in amount or level, did the study examine different levels of the exposure as related to the outcome (e.g., categories of exposure, or exposure measured as continuous variable)?	Yes	No	No	Yes	No	Yes	No	Yes
Were the exposure measures (independent variables) clearly defined, valid, reliable, and implemented consistently across all study participants?	Yes	No	No	Yes	No	Yes	Yes	Yes
Was the exposure(s) assessed more than once over time?	No	No	No	No	No	No	No	No
Were the outcome measures (dependent variables) clearly defined, valid, reliable, and implemented consistently across all study participants?	Yes	Yes	Yes	Yes	Yes	Yes	Yes	Yes
Were the outcome assessors blinded to the exposure status of participants?	No	No	No	No	No	No	No	No
Was loss to follow-up after baseline 20% or less?	Yes	Yes	Yes	Yes	Yes	Yes	Yes	Yes
Were key potential confounding variables measured and adjusted statistically for their impact on the relationship between exposure(s) and outcome(s)?	Yes	Yes	Yes	Yes	Yes	Yes	Yes	Yes
**Quality Rating**	Good	Low	Fair	Good	Low	Good	Good	Good
**Risk of Bias**	Low	Moderate	Unclear	Low	Moderate	Low	Low	Low

CD, cannot determine; NA, not applicable; NR, not reported

Table 2A: Cohort studies (continued)

Criteria	Ibrahim Sahin et al[91]	Jayanama et al[87]	Kananen et al[180]	Kang et al[89]	Kompaniyets et al[90]	Emma J. Kooistra et al[184]	Emma J. Kooistra et al-2[183]	Larvin et al[185]
Was the research question or objective in this paper clearly stated?	Yes	Yes	Yes	Yes	Yes	Yes	Yes	Yes
Was the study population clearly specified and defined?	Yes	Yes	Yes	Yes	Yes	Yes	Yes	Yes
Was the participation rate of eligible persons at least 50%?	Yes	NR	Yes	Yes	Yes	Yes	NR	Yes
Were all the subjects selected or recruited from the same or similar populations (including the same time period)? Were inclusion and exclusion criteria for being in the study prespecified and applied uniformly to all participants?	Yes	Yes	Yes	Yes	Yes	Yes	Yes	Yes
Was a sample size justification, power description, or variance and effect estimates provided?	NR	NR	NR	NR	NR	NR	NR	NR
For the analyses in this paper, was the exposure(s) of interest measured prior to the outcome(s) being measured?	Yes	Yes	Yes	Yes	Yes	Yes	Yes	Yes
Was the timeframe sufficient so that one could reasonably expect to see an association between exposure and outcome if it existed?	Yes	Yes	Yes	Yes	Yes	Yes	Yes	Yes
For exposures that can vary in amount or level, did the study examine different levels of the exposure as related to the outcome (e.g., categories of exposure, or exposure measured as continuous variable)?	Yes	Yes	Yes	Yes	Yes	No	Yes	Yes
Were the exposure measures (independent variables) clearly defined, valid, reliable, and implemented consistently across all study participants?	Yes	Yes	Yes	Yes	Yes	CD	Yes	Yes
Was the exposure(s) assessed more than once over time?	No	No	No	No	No	No	No	No
Were the outcome measures (dependent variables) clearly defined, valid, reliable, and implemented consistently across all study participants?	Yes	Yes	Yes	Yes	Yes	Yes	Yes	Yes
Were the outcome assessors blinded to the exposure status of participants?	No	No	No	No	No	No	No	No
Was loss to follow-up after baseline 20% or less?	Yes	Yes	Yes	Yes	Yes	Yes	Yes	Yes
Were key potential confounding variables measured and adjusted statistically for their impact on the relationship between exposure(s) and outcome(s)?	Yes	Yes	Yes	Yes	Yes	No	Yes	Yes
**Quality Rating**	Good	Good	Good	Good	Good	Low	Good	Good
**Risk of Bias**	Low	Low	Low	Low	Low	Moderate	Low	Low

CD, cannot determine; NA, not applicable; NR, not reported

Table 2A: Cohort studies (continued)

Criteria	Le Guen et al[96]	Martín-Del-Campo et al[99]	Mehanna et al[100]	Mehta et al[187]	Min Gao et al[93]	Naaraayan et al[191]	Nam Hoon Kim et al[108]	Okauchi et al[110]
Was the research question or objective in this paper clearly stated?	Yes	Yes	Yes	Yes	Yes	Yes	Yes	Yes
Was the study population clearly specified and defined?	Yes	Yes	Yes	Yes	Yes	Yes	Yes	Yes
Was the participation rate of eligible persons at least 50%?	Yes	Yes	NR	Yes	Yes	Yes	Yes	Yes
Were all the subjects selected or recruited from the same or similar populations (including the same time period)? Were inclusion and exclusion criteria for being in the study prespecified and applied uniformly to all participants?	Yes	Yes	Yes	Yes	Yes	Yes	Yes	Yes
Was a sample size justification, power description, or variance and effect estimates provided?	NR	NR	NR	NR	NR	NR	NR	NR
For the analyses in this paper, was the exposure(s) of interest measured prior to the outcome(s) being measured?	Yes	Yes	Yes	Yes	Yes	Yes	Yes	Yes
Was the timeframe sufficient so that one could reasonably expect to see an association between exposure and outcome if it existed?	Yes	Yes	Yes	Yes	Yes	No	Yes	No
For exposures that can vary in amount or level, did the study examine different levels of the exposure as related to the outcome (e.g., categories of exposure, or exposure measured as continuous variable)?	Yes	Yes	Yes	Yes	Yes	Yes	Yes	No
Were the exposure measures (independent variables) clearly defined, valid, reliable, and implemented consistently across all study participants?	Yes	Yes	Yes	Yes	Yes	Yes	Yes	Yes
Was the exposure(s) assessed more than once over time?	No	No	No	Yes	Yes	No	No	No
Were the outcome measures (dependent variables) clearly defined, valid, reliable, and implemented consistently across all study participants?	Yes	Yes	Yes	Yes	Yes	Yes	Yes	Yes
Were the outcome assessors blinded to the exposure status of participants?	No	No	No	No	No	No	No	No
Was loss to follow-up after baseline 20% or less?	Yes	Yes	Yes	Yes	Yes	Yes	Yes	Yes
Were key potential confounding variables measured and adjusted statistically for their impact on the relationship between exposure(s) and outcome(s)?	Yes	Yes	Yes	Yes	Yes	Yes	Yes	Yes
**Quality Rating**	Good	Good	Good	High	High	Good	Good	Fair
**Risk of Bias**	Low	Low	Low	Very Low	Very Low	Low	Low	Unclear

CD, cannot determine; NA, not applicable; NR, not reported

Table 2A: Cohort studies (continued)

Criteria	Olak et al[192]	Olivas-Martínez et al[193]	Oliveira et al[194]	Page-Wilson et al[112]	Patel et al[197]	Peña et al[198]	Pietri et al[118]	Plataki et al[119]
Was the research question or objective in this paper clearly stated?	Yes	Yes	Yes	Yes	Yes	Yes	Yes	Yes
Was the study population clearly specified and defined?	Yes	Yes	Yes	Yes	Yes	Yes	Yes	Yes
Was the participation rate of eligible persons at least 50%?	Yes	Yes	Yes	Yes	Yes	Yes	Yes	Yes
Were all the subjects selected or recruited from the same or similar populations (including the same time period)? Were inclusion and exclusion criteria for being in the study prespecified and applied uniformly to all participants?	Yes	Yes	Yes	Yes	Yes	Yes	Yes	Yes
Was a sample size justification, power description, or variance and effect estimates provided?	NR	NR	NR	NR	NR	NR	NR	NR
For the analyses in this paper, was the exposure(s) of interest measured prior to the outcome(s) being measured?	Yes	Yes	Yes	Yes	Yes	Yes	Yes	Yes
Was the timeframe sufficient so that one could reasonably expect to see an association between exposure and outcome if it existed?	No	No	No	Yes	Yes	Yes	No	Yes
For exposures that can vary in amount or level, did the study examine different levels of the exposure as related to the outcome (e.g., categories of exposure, or exposure measured as continuous variable)?	No	Yes	No	Yes	Yes	No	Yes	Yes
Were the exposure measures (independent variables) clearly defined, valid, reliable, and implemented consistently across all study participants?	No	Yes	No	Yes	Yes	No	Yes	Yes
Was the exposure(s) assessed more than once over time?	No	No	No	No	No	No	No	No
Were the outcome measures (dependent variables) clearly defined, valid, reliable, and implemented consistently across all study participants?	Yes	Yes	Yes	Yes	Yes	Yes	Yes	Yes
Were the outcome assessors blinded to the exposure status of participants?	No	No	No	No	No	No	No	No
Was loss to follow-up after baseline 20% or less?	Yes	Yes	Yes	Yes	Yes	Yes	Yes	Yes
Were key potential confounding variables measured and adjusted statistically for their impact on the relationship between exposure(s) and outcome(s)?	Yes	Yes	Yes	Yes	Yes	Yes	Yes	Yes
**Quality Rating**	Low	Good	Low	Good	Good	Fair	Good	Good
**Risk of Bias**	Moderate	Low	Moderate	Low	Low	Unclear	Low	Low

CD, cannot determine; NA, not applicable; NR, not reported

Table 2A: Cohort studies (continued)

Criteria	Plourde et al[120]	Pouwels et al[122]	Randhawa et al[126]	Recalde et al[128]	Rodriguez-Nava et al[202]	Saito et al[133]	Sardinha et al[204]	Schavemaker et al[206]
Was the research question or objective in this paper clearly stated?	Yes	Yes	Yes	Yes	Yes	Yes	Yes	Yes
Was the study population clearly specified and defined?	Yes	Yes	Yes	Yes	Yes	Yes	Yes	Yes
Was the participation rate of eligible persons at least 50%?	NR	Yes	No	Yes	Yes	Yes	Yes	Yes
Were all the subjects selected or recruited from the same or similar populations (including the same time period)? Were inclusion and exclusion criteria for being in the study prespecified and applied uniformly to all participants?	Yes	Yes	Yes	Yes	Yes	Yes	Yes	Yes
Was a sample size justification, power description, or variance and effect estimates provided?	NR	No	Yes	NR	NR	NR	NR	No
For the analyses in this paper, was the exposure(s) of interest measured prior to the outcome(s) being measured?	Yes	Yes	Yes	Yes	Yes	Yes	No	Yes
Was the timeframe sufficient so that one could reasonably expect to see an association between exposure and outcome if it existed?	No	No	No	Yes	No	No	No	Yes
For exposures that can vary in amount or level, did the study examine different levels of the exposure as related to the outcome (e.g., categories of exposure, or exposure measured as continuous variable)?	No	No	No	No	No	Yes	No	Yes
Were the exposure measures (independent variables) clearly defined, valid, reliable, and implemented consistently across all study participants?	No	No	No	No	No	Yes	No	Yes
Was the exposure(s) assessed more than once over time?	No	No	Yes	No	No	No	No	No
Were the outcome measures (dependent variables) clearly defined, valid, reliable, and implemented consistently across all study participants?	Yes	Yes	Yes	Yes	Yes	Yes	Yes	Yes
Were the outcome assessors blinded to the exposure status of participants?	No	No	No	No	No	No	No	No
Was loss to follow-up after baseline 20% or less?	Yes	Yes	Yes	Yes	Yes	Yes	Yes	Yes
Were key potential confounding variables measured and adjusted statistically for their impact on the relationship between exposure(s) and outcome(s)?	Yes	Yes	Yes	No	Yes	Yes	Yes	Yes
**Quality Rating**	Low	Low	Fair	Low	Low	Good	Low	Good
**Risk of Bias**	Moderate	Moderate	Unclear	Moderate	Moderate	Low	Moderate	Low

CD, cannot determine; NA, not applicable; NR, not reported

Table 2A: Cohort studies (continued)

Criteria	Serdar Sahin et al[132]	Shaikh et al[134]	Silva et al[136]	So Young Kim et al[124]	Suresh et al[141]	Tara S. Kim et al[138]	Tchang et al[142]	Tehrani et al[207]
Was the research question or objective in this paper clearly stated?	Yes	Yes	Yes	Yes	Yes	Yes	Yes	Yes
Was the study population clearly specified and defined?	Yes	Yes	Yes	Yes	Yes	Yes	Yes	Yes
Was the participation rate of eligible persons at least 50%?	Yes	Yes	Yes	Yes	Yes	Yes	Yes	No
Were all the subjects selected or recruited from the same or similar populations (including the same time period)? Were inclusion and exclusion criteria for being in the study prespecified and applied uniformly to all participants?	Yes	Yes	Yes	Yes	Yes	Yes	Yes	Yes
Was a sample size justification, power description, or variance and effect estimates provided?	NR	NR	NR	NR	NR	NR	NR	NR
For the analyses in this paper, was the exposure(s) of interest measured prior to the outcome(s) being measured?	No	Yes	Yes	Yes	Yes	Yes	Yes	Yes
Was the timeframe sufficient so that one could reasonably expect to see an association between exposure and outcome if it existed?	No	No	Yes	Yes	Yes	Yes	Yes	No
For exposures that can vary in amount or level, did the study examine different levels of the exposure as related to the outcome (e.g., categories of exposure, or exposure measured as continuous variable)?	Yes	Yes	Yes	Yes	Yes	Yes	Yes	No
Were the exposure measures (independent variables) clearly defined, valid, reliable, and implemented consistently across all study participants?	Yes	No	Yes	Yes	No	Yes	Yes	No
Was the exposure(s) assessed more than once over time?	No	No	No	No	No	No	No	No
Were the outcome measures (dependent variables) clearly defined, valid, reliable, and implemented consistently across all study participants?	Yes	Yes	Yes	Yes	Yes	Yes	Yes	Yes
Were the outcome assessors blinded to the exposure status of participants?	No	No	No	No	No	No	No	No
Was loss to follow-up after baseline 20% or less?	NA	No	No	Yes	Yes	Yes	Yes	Yes
Were key potential confounding variables measured and adjusted statistically for their impact on the relationship between exposure(s) and outcome(s)?	Yes	Yes	Yes	Yes	Yes	Yes	Yes	Yes
**Quality Rating**	Low	Low	Good	Good	Good	Good	Good	Low
**Risk of Bias**	Moderate	Moderate	Low	Low	Low	Low	Low	Moderate

CD, cannot determine; NA, not applicable; NR, not reported

Table 2A: Cohort studies (continued)

Criteria	Terada et al[143]	Vera-Zertuche et al[147]	Wu et al[149]	Yates et al[153]	Zhang et al[155]	Zhu et al[154]
Was the research question or objective in this paper clearly stated?	Yes	Yes	Yes	Yes	Yes	Yes
Was the study population clearly specified and defined?	Yes	Yes	Yes	Yes	Yes	Yes
Was the participation rate of eligible persons at least 50%?	Yes	Yes	Yes		Yes	NR
Were all the subjects selected or recruited from the same or similar populations (including the same time period)? Were inclusion and exclusion criteria for being in the study prespecified and applied uniformly to all participants?	Yes	Yes	Yes	Yes	Yes	Yes
Was a sample size justification, power description, or variance and effect estimates provided?	NR	Yes	NR	NR	NR	NR
For the analyses in this paper, was the exposure(s) of interest measured prior to the outcome(s) being measured?	Yes	Yes	Yes	Yes	No	Yes
Was the timeframe sufficient so that one could reasonably expect to see an association between exposure and outcome if it existed?	Yes	Yes	Yes	Yes	No	Yes
For exposures that can vary in amount or level, did the study examine different levels of the exposure as related to the outcome (e.g., categories of exposure, or exposure measured as continuous variable)?	No	No	Yes		Yes	Yes
Were the exposure measures (independent variables) clearly defined, valid, reliable, and implemented consistently across all study participants?	No	No	Yes		Yes	Yes
Was the exposure(s) assessed more than once over time?	No	Yes	No	No	No	No
Were the outcome measures (dependent variables) clearly defined, valid, reliable, and implemented consistently across all study participants?	Yes	Yes	Yes	Yes	Yes	Yes
Were the outcome assessors blinded to the exposure status of participants?	No	No	No	No	No	No
Was loss to follow-up after baseline 20% or less?	Yes	Yes	Yes		NR	NA
Were key potential confounding variables measured and adjusted statistically for their impact on the relationship between exposure(s) and outcome(s)?	NR	Yes	Yes		Yes	Yes
**Quality Rating**	Low	Good	Good	Very Low	Low	Fair
**Risk of Bias**	Moderate	Low	Low	High	Moderate	Unclear

CD, cannot determine; NA, not applicable; NR, not reported

Table 2B: Case control studies

Criteria	Urra *et al.*[145]
Was the research question or objective in this paper clearly stated and appropriate?	Yes
Was the study population clearly specified and defined?	Yes
Did the authors include a sample size justification?	NR
Were controls selected or recruited from the same or similar population that gave rise to the cases (including the same timeframe)?	Yes
Were the definitions, inclusion and exclusion criteria, algorithms, or processes used to identify or select cases and controls valid, reliable, and implemented consistently across all study participants?	Yes
Were the cases clearly defined and differentiated from controls?	Yes
If less than 100 percent of eligible cases and/or controls were selected for the study, were the cases and/or controls randomly selected from those eligible?	NA
Was there use of concurrent controls?	NA
Were the investigators able to confirm that the exposure/risk occurred prior to the development of the condition or event that defined a participant as a case?	NA
Were the measures of exposure/risk clearly defined, valid, reliable, and implemented consistently (including the same time period) across all study participants?	Yes
Were the assessors of exposure/risk blinded to the case or control status of participants?	NR
Were key potential confounding variables measured and adjusted statistically in the analyses? If matching was used, did the investigators account for matching during study analysis?	Yes
**Quality Rating**	
**Risk of Bias**	

CD, cannot determine; NA, not applicable; NR, not reported

Table 2C: Case series

Criteria	Bhatraju *et al*[53]
Was the study question or objective clearly stated?	Yes
Was the study population clearly and fully described, including a case definition?	Yes
Were the cases consecutive?	Yes
Were the subjects comparable?	Yes
Was the intervention clearly described?	NA
Were the outcome measures clearly defined, valid, reliable, and implemented consistently across all study participants?	Yes
Was the length of follow-up adequate?	No
Were the statistical methods well-described?	Yes
Were the results well-described?	Yes
**Quality Rating**	Good
**Risk of Bias**	Low risk of bias but have some potential flaws

CD, cannot determine; NA, not applicable; NR, not reported
